# Inhibition of CDC27 O-GlcNAcylation coordinates the antitumor efficacy in multiple myeloma through the autophagy-lysosome pathway

**DOI:** 10.1038/s41401-025-01500-2

**Published:** 2025-02-21

**Authors:** Hai-qi Wu, Ren-cai Qin, Wei-jie Li, Jie-na Liu, Chong Deng, Zi-han Zheng, Jing-peng Zheng, Yu Liu, Yan-fang Meng, Chun Tang, Hong-mei Tan, Fang-fang Duan, Yuan Tang, Fan Xiao, Li-wei Lu, Xiao-yan Dai, Kong-yang Ma

**Affiliations:** 1 Shenzhen Key Laboratory for Systems Medicine in Inflammatory Diseases, Centre for Infection and Immunity Studies (CIIS), School of Medicine, Shenzhen Campus of Sun Yat-sen University, Shenzhen, 518107 China; 2 Department of Pathology, The University of Hong Kong, Hong Kong, China; 3 Centre for Oncology and Immunology, Hong Kong Science Park, Hong Kong, China; 4 Center for Infectious Disease Research, School of Medicine, Westlake University, Hangzhou, 310000 China; 5 Center of Kidney and Urology, The Seventh Affiliated Hospital, Shenzhen, 518107 China; 6 Clinical Research Institute, The Second Affiliated Hospital, University of South China, Hengyang, 421002 China

**Keywords:** multiple myeloma, machine learning, O-GlcNAcylation, CDC27, bortezomib

## Abstract

Multiple myeloma (MM) is a prevalent hematologic malignancy characterized by abnormal proliferation of cloned plasma cells. Given the aggressive nature and drug resistance of MM cells, identification of novel genes could provide valuable insights for treatment. In this study we performed machine learning in the RNA microarray data of purified myeloma plasma cell samples from five independent MM cohorts with 957 MM patients, and identified O-GlcNAcylation transferase (*OGT*) and cell division cycle *27* (*CDC27*) as the key prognostic genes for MM. We demonstrated a close link between OGT and CDC27 in MM cells by knockdown of OGT with siOGT, pharmacological inhibition of O-GlcNAcylation with OSMI-1 and pharmacological accumulation of O-GlcNAcylation with Thiamet G. Using mass spectrometry and immunoprecipitation, we identified the O-GlcNAcylated CDC27 protein as a key target protein that may be directly downregulated by OSMI-1 in MM.1S cells. We further revealed that O-GlcNAcylation maintained CDC27 protein stability by blocking the autophagy-lysosome pathway (ALP). Moreover, we demonstrated the enhanced antitumor efficacy of combined OSMI-1 and bortezomib (BTZ) treatment in MM cells both in vivo and in vitro. Thus, this study identifies a novel function of O-GlcNAcylation-related ALP in regulating CDC27 protein stability and a potential therapeutic strategy for treating MM.

## Introduction

Multiple myeloma (MM) is the second most common hematologic malignancy characterized by abnormal proliferation of cloned plasma cells, secretion of monoclonal immunoglobulins, and associated multiple organ dysfunction [1]. Although treatments for MM with improved efficacy have been reported, drug resistance is becoming a great challenge in patients with relapsed refractory MM. For instance, the use of bortezomib (BTZ) in MM patients is limited by the drug resistance and disease malignant features [2, 3]. Previous studies indicated the low expression of XBP1, ATF3, and ATF4 in poor responders to BTZ treatment [4]. We previously characterized the phenotypic and functional features of pathogenic plasma cells in autoimmune disease [5]. Moreover, a recent study utilized single-cell RNA sequencing to characterize the specific features associated with BTZ resistance in MM patients, highlighting the roles of MM-specific natural killer (NK) cells and T cells in determining responsiveness to BTZ treatment [6]. Even though several reported diagnostic biomarkers in MM have been identified [7, 8], further screening and characterization of novel diagnostic biomarkers for MM using advanced methods may facilitate the early diagnosis and therapeutic intervention of MM patients.

In recent years, advancements in microarray technology have improved its accuracy and efficiency, enabling extensive analysis of disease-related databases. Progress in bioinformatics has also enhanced biomedical discoveries. Machine learning is a powerful classification tool capable of extracting high-dimensional features from large disease-related datasets [9]. Moreover, large-scale patient cohorts are required to mitigate false-negative outcomes associated with limited sample sizes. The application of machine learning for screening and identifying effective, novel diagnostic biomarkers for MM could enhance early diagnosis and inform therapeutic intervention for MM patients.

Current studies have shown that the pathogenesis of drug resistance of MM may involve genetic factors and metabolic reprogramming, such as enhanced glycolysis [7, 8, 10]. Abnormal glucose uptake in tumor cells fuels O-GlcNAcylation, which is a dynamic and reversible protein posttranslational modification [11, 12]. Previous studies have revealed a positive correlation between O-GlcNAcylation and tumor growth via the stabilization of target proteins such as phosphoglycerate kinase 1 (PGK1) and the ribosomal receptor for activated C-kinase 1 (RACK1) [13, 14]. Importantly, O-GlcNAcylation transferase (OGT) is the sole enzyme capable of catalyzing the addition of O-GlcNAcylation to target proteins [11]. O-GlcNAcylation has been reported to be crucial for posttranscriptional modulation of the germinal center B-cell response and antibody secretion in B-cell-specific *Ogt*-deficient mice [15]. O-GlcNAcylation modifications can occur on specific amino acids to control protein stability. OSMI-1 is a novel, small cell-permeable molecule that inhibits OGT. Previous studies have reported that OSMI-1 can modulate the function of tumor cells. However, whether metabolic reprogramming-related enzyme activity of OGT is involved in the progression and drug resistance of MM remains largely unclear.

In this study, we first identified O-GlcNAcylation transferase (OGT) as a key hub gene for MM pathogenesis via three machine learning models based on the RNA microarray data of purified myeloma plasma cell samples from five independent MM cohorts with 957 MM patients. Then, we investigated the effect and underlying mechanism of O-GlcNAcylation on the survival and proliferation of MM cells with pharmacological inhibition, accumulation of O-GlcNAcylation (OSMI-1 and Thiamet G) and knockdown of the *OGT* gene. Notably, we identified CDC27 as a protein targeted by OGT-mediated glycosylation via mass spectrometry and immunoprecipitation analysis. Knockdown of *CDC27* abrogated the O-GlcNAcylation accumulation-induced MM cell survival. Further mechanistic studies revealed that O-GlcNAcylation directly controls the protein stability of CDC27, with OSMI-1-reduced the CDC27 protein stability and Thiamet G-prolonged protein stability of CDC27 via activating the autophagy-lysosome pathway (ALP). Moreover, we demonstrated the enhanced therapeutic effect of the OGT inhibitor OSMI-1 combined with BTZ on MM cells both in vitro and in vivo. Together, our findings revealed a novel function of O-GlcNAcylation-related ALP in regulating CDC27 protein stability and identified a potential therapeutic target for treating MM.

## Materials and methods

### Differential gene analysis

The transcriptome profiles and clinical data for MM (GSE5900, GSE19784, and GSE136337) were acquired from the Gene Expression Omnibus (GEO) database (https://www.ncbi.nlm.nih.gov/geo/). The R package “*limma*” was used to identify genes with significant differences between MM patients and healthy individuals. By setting the threshold |Log2-Fold Change (FC)| ≥ 0.5, significantly up- or downregulated genes were screened out, and these genes were subsequently used as the feature gene set input for the machine learning model.

### Feature selection

The elastic net logistic regression method, implemented in the “*glmnet*” package, was used to further refine the feature gene set in this study. This approach merged the L1 and L2 penalty terms, effectively addressing multicollinearity and feature selection issues, thereby enhancing the predictive accuracy and interpretability of the model.

### Machine learning modeling

Based on the selected characteristic genes, three different machine learning algorithms, gradient boosting machine (the “*gbm*” package), random forest (the “*randomForest*” package), and extreme gradient boosting (the “*xgboost*” package), were used for model construction and training. Each method was run independently, and the characteristic genes common to each model were extracted as the hub genes.

### Performance evaluation and validation

The “*pROC*” package was used to generate the receiver operating characteristic (ROC) curves of each machine learning model, and the corresponding area under the curve (AUC) value was calculated to evaluate the classification performance of the models. In addition, the selected hub gene set was validated on independent training and test sets in this study to evaluate its generalizability and stability.

### Kaplan-Meier survival analysis

The relationship between OGT expression levels and overall survival (OS) in multiple myeloma (MM) patients was thoroughly examined through Kaplan-Meier survival analysis coupled with the log-rank test. This analytical approach allowed for the detailed assessment of survival probabilities over time, providing a robust statistical framework to determine whether variations in OGT expression significantly impacted patient outcomes.

### Correction for batch effects

To mitigate batch effects in the gene expression data (Supplementary Table S1), we utilized the ComBat function from the sva R package. Prior to the correction, the data were log_2_-transformed. Post ComBat, we assessed batch effect removal using principal component analysis, ensuring that batch-related variability was minimized while preserving biological distinctions.

### Gene set enrichment analysis

To evaluate whether there were statistically significant differences in the expression of specific gene sets between multiple myeloma patients and healthy individuals, the gene set enrichment analysis (GSEA) method was performed. Briefly, gene expression data were first sorted according to their association with phenotypes, and then the enrichment scores (ES) of predefined gene sets were calculated to determine whether gene sets were enriched at the top and bottom. The gseKEGG and gseGO functions in the clusterProfiler package were used to perform GSEA enrichment analysis.

### Main reagents

OSMI-1, Thiamet G, bortezomib, cycloheximide, 3-methyladenine, and chloroquine were purchased from MedChemExpress (Monmouth Junction, NJ, USA). The chemical structure of OSMI-1 was depicted via the ChemDraw software. Purified anti-O-GlcNAcylation (CTD 110.6), purified mouse IgG1 isotype control, biotin mouse anti-human CD138, PE/cyanine5 streptavidin, Percp/cy7 anti-human CD27, Violet 421^TM^ anti-human CD19, FITC Annexin-V, Zombie Aqua™, were acquired from Biolegend (San Diego, CA, USA). Anti-OGT antibody was purchased from Abcam (Cambridge, UK), and anti-O-GlcNAcylation (RL2) antibody was purchased from Invitrogen (Carlsbad, CA, USA). Additionally, anti-CDC27, anti-β-actin, anti-LAMP1, anti-PSMB5, anti-p62, anti-ubiquitin, rabbit IgG control, R-PE-conjugated goat anti-rabbit IgG (H + L), and coraLite-488-conjugated goat anti-rabbit/mouse IgG (H + L) antibodies were purchased from Proteintech (Wuhan, China). Anti-LC3B was purchased from Cell Signaling Technology (Danvers, MA, USA). Hoechst 33342 was obtained from Beyotime (Shanghai, China). Lysotracker was obtained from Yeasen (Shanghai, China).

### Cell culture

The MM cell line MM.1S was purchased from Guangzhou Xinyuan Biotechnology Co., Ltd. Cells were cultured in RPMI-1640 media (Procell, Wuhan, China) supplemented with 10% fetal bovine serum (Excell, Suzhou, China), 100 U/mL penicillin, 100 μg/mL streptomycin, 2 mM *L*-glutamine (Gibco, Grand Island, NY, USA) and 55 µM β-mercaptoethanol (Procell) at 37 °C with 5% CO_2_.

### Cell isolation

Human PBMCs were obtained from Leide Biosciences Co., Ltd. (Guangzhou, China). Human CD138^+^ plasma cells were isolated via the immunomagnetic positive selection. PBMCs were resuspended in MACS buffer and incubated with a biotin-conjugated anti-human CD138 antibody at 1:50 for 20 min, followed by centrifugation. The precipitate was resuspended and incubated with streptavidin microbeads (RWD Life Science, Shenzhen, China) at a 1:50 ratio for 20 min. Subsequently, magnetic separation yielded purified human CD138^+^ plasma cells according to the manufacturer’s instructions (RWD Life Science).

### Transfection of siRNA

MM.1S cells were transfected with 30 nM of OGT or CDC27 siRNA using Neon Transfection System (Invitrogen) and the scrambled siRNA sequence was used as the negative control (Sangon Biotech, Shanghai, China). MM cell transfection was performed using antibiotic free RPMI-1640 media and the indicated condition (Voltage - 1500 V, Width - 10 ms, Pulses - 3). After 48 h post-transfection, cells were harvested or subjected to additional treatments. The siRNA sequences used for the transfection were listed in Supplementary Table S2.

### Animal models

NSG mice (female, 6–8 weeks) were purchased from Caverns Experimental Animal Co., Ltd. (Changzhou, China) and used at Shenzhen TopBiotech Co., Ltd. (Shenzhen, China). The study was approved and conducted following the guidelines of the Institutional Review Board (IRB) of Shenzhen TopBiotech Co., Ltd. All experiments were performed in compliance with institutional animal care guidelines and protocols approved by the Animal Research Ethics Committees of TopBiotech Co., Ltd. (number: TOPGM-IACUC-2023-0124). A total of 5 × 10^6^ MM.1S cells were intravenously injected into NCG mice to establish a disseminated human MM xenograft model as previously reported [16]. At 2 weeks post cell transfer, OSMI-1 (5 mg/kg) was injected intraperitoneally daily for 2 weeks, and then BTZ (0.5 mg/kg) was injected intraperitoneally twice a week. Human MM cells in the abdominal cavity and bone marrow were stained with the live/dead dye (Zombie Aqua™) and Fc Receptor Blocking Solution (Human TruStain FcX™, BioLegend). Then, the biotin mouse anti-human CD138 antibody and PE/cyanine5 streptavidin (1:200) were used for human CD138 staining. Cell membrane was permeabilized with the True-Nuclear™ Transcription Factor Buffer Set (BioLegend) and then stained with anti-CDC27 antibody and PE-goat anti-rabbit antibody (1:300) overnight at 4 °C. The human CD138^+^ and CDC27^+^ cells were detected on a Beckman Coulter flow cytometer (Miami, FL, USA) and analyzed via the FlowJo software (Becton, Dickinson & Company, Franklin Lakes, NJ, USA).

### Quantitative real-time PCR (qRT-PCR)

MM cells were plated in 6-well plates at a density of 5 × 10^6^ cells/well and then treated with 30 µM OSMI-1 or 10 nM BTZ for 24 h. Total RNA was extracted via TRIzol and then subjected to complementary DNA (cDNA) synthesis via an Evo M-MLV RT Mix Kit (Accurate Biology, Changsha, China). The following qPCR was conducted with a SYBR Green Premix Pro Taq HS qPCR kit (AG). qPCR was performed via the QuantStudio 5 Real-Time PCR System (Thermo Fisher Scientific, Waltham, MA, USA). The relative expression of genes was analyzed via the 2^−∆∆Ct^ method with β-actin as an internal reference. The primers used for the qPCR analysis were listed in Supplementary Table S2.

### Western blotting

MM.1S cells were plated in 6-well plates at a density of 5 × 10^6^ cells/well and then subjected to indicated treatment. Whole-cell lysates were extracted from the RIPA-lysate (CWBIO, Taizhou, China) supplemented with phosphatase inhibitor cocktail (CWBIO) and quantitatively analyzed with a BCA kit (CWBIO). The extracted protein was subsequently separated through a 12% sodium dodecyl sulfate-polyacrylamide gel and transferred onto polyvinylidene fluoride membranes (PVDF, Merck Millipore, Billerica, MA, USA). The PVDF membranes were blocked in 5% fat-free milk (O-GlcNAcylation and LC3B in 5% BSA) for 1.5 h, incubated with primary antibodies at 4 °C overnight, and then incubated with secondary antibodies for 1 h at RT. Protein signals were examined via an enhanced chemiluminescence kit (GlpBio, Montclair, CA, USA). The gray values of each protein band were analyzed via ImageJ software (National Institutes of Health, Bethesda, MD, USA).

### Immunoprecipitation (IP)

Whole-cell lysates were extracted with cell lysis buffer for Western blotting and IP (Beyotime). Specific antibodies (RL2 or anti-CDC27 antibody) and isotype control antibody (purified mouse IgG1 or rabbit IgG) were incubated with rProtein A/G beads 4FF (Smart-Lifesciences, Changzhou, China) at 4 °C for 4 h. Cell lysates were first incubated with unprocessed beads for 2 h to eliminate nonspecific binding, and the supernatant obtained by centrifugation was quantified by the BCA assay. The sample was boiled at 100 °C for 10 min and verified by SDS-PAGE. Differences in O-GlcNAcylated proteins were assessed by Coomassie blue staining (Biosharp, Hefei, China) for SDS‒PAGE.

### Liquid chromatography-tandem mass spectrometry (LC-MS/MS)

Mass spectrometry analysis was conducted by SinoGenoMax Biotechnologies Co., Ltd. (Shanghai, China). The gel sample was washed with 200–400 μL of decolorizing solution until it became transparent. After the supernatant was discarded, the sample was freeze-dried and incubated with DTT and IAA in sequence. The sample was subsequently washed with ACN and freeze-dried again before the addition of trypsin solution, after which the mixture was allowed to react for 20 h. The enzymatic hydrolysate was then transferred to a new EP tube. An extraction mixture was added to the gel for ultrasonic extraction, and the combined mixture was freeze-dried, resuspended, desalted, and then resuspended again in preparation for mass spectrometry analysis. The sample was separated via the nanoliter-flow HPLC system Easy nLC, which employs 0.1% formic acid aqueous solution and acetonitrile solution as buffers. A chromatographic column was used for separation at a flow rate of 300 nL/min. After separation, a Q Exactive mass spectrometer was used for mass spectrometry analysis, with a duration of 120 min. Positive ion detection was employed, with a parent ion scan range of 300–1800 *m*/*z*. The first-order mass spectrometry resolution was set at 70,000, with an AGC target of 1e6. Peptide fragment acquisition was carried out via the HCD method, with a second-order mass spectrometry resolution of 17,500 and an AGC target of 1e5. The raw files of mass spectrometry tests were searched against databases via MaxQuant 1.6.14 for the identification of proteins.

### Immunofluorescence staining

A total of 1 × 10^6^ cells were fixed and permeabilized with cold acetone, followed by blocking with 5% BSA for 1 h at RT. The cells were incubated with primary antibodies (anti-OGT and anti-CDC27 antibodies 1:250, anti-O-GlcNAcylation antibody 1:100, anti-LC3B and anti-LAMP1 antibodies 1:200) overnight at 4 °C, followed by incubation with the corresponding secondary antibodies (1:500) for 1 h at RT. Hoechst stain (1:1000) was applied for 10 min at RT. Images were acquired via a Zeiss LSM900 confocal microscope (Jena, Germany), and analysis was performed via the ZEN (Zeiss, Jena, Germany) and ImageJ software.

### Protein stability assay

MM.1S cells were pretreated with cycloheximide (CHX, 100 µg/mL) for 30 min before being treated with or without 30 µM OSMI-1 or 50 nM Thiamet G for the indicated time points, respectively. Western blot analysis was then conducted to detect the half-life of CDC27 protein degradation.

Additionally, MM.1S cells were treated with the proteasome inhibitor BTZ (10 nM), the lysosome inhibitor CQ (25 µM), or the autophagy inhibitor 3-MA (1 mM) in the presence or absence of 30 µM OSMI-1 treatment for 24 h, and CDC27 levels were detected by Western blot analysis.

### Cell viability assay

MM cells were plated in 96-well plates at a density of 5×10^4^ cells/well in 200 µL culture medium system and then subjected to indicated treatment. Subsequently, 20 µL of CCK-8 reagent (GlpBio) was added, followed by incubation at 37 °C for 1 h. The absorbance of each well was measured at 450 nm on a BIOTEK Synergy H1 Microplate Reader (Agilent Technologies, Santa Clara, CA, USA).

### Drug combination analysis

MM.1S cells were treated with an indicated dose of OSMI-1 and BTZ in monotherapy and combination for 24 h. The combination treatments were performed maintaining a constant ratio (3:1) between the dose of OSMI-1 and BTZ. Synergism between OSMI-1 and BTZ was evaluated using the Chou-Talalay method [17]. Fraction affected (FA) versus the combination index (CI) plot was drawn using the CalcuSyn (Biosoft). CI < 1, CI = 1, and CI > 1 indicate synergistic, additive, and antagonistic effects, respectively.

### Cell apoptosis assay

MM cells were plated in 48-well plates at a density of 5 × 10^5^ cells/well and then subjected to indicated treatment. After treatment, the cells were washed once with PBS and stained with Zombie Aqua™ (1:1000) for 10 min at RT. The cells were subsequently stained with Annexin V (1:100) for 1 h at RT. All samples were analyzed via a Beckman Coulter flow cytometer and analyzed via the FlowJo software (Becton).

### Cell cycle analysis

The cell cycle distribution was measured via a cell cycle detection kit (Keygen Biotech, Nanjing, China). MM.1S cells were washed once with PBS and fixed with 70% cold ethanol at 4 °C overnight. After removing the fixing solution, the cells were stained with 500 µL of PI/RNase A staining solution (9:1) and incubated at RT for 30 min. All samples were analyzed via a Beckman Coulter flow cytometer and analyzed via the FlowJo software (Becton).

### Statistical analysis

The results are presented as the means ± standard deviation (SD). Statistical analysis was performed via GraphPad Prism (Version X; La Jolla, CA, USA), which employs a two-tailed unpaired Student’s *t*-test, and two-tailed Mann-Whitney U test as indicated in comparison between two groups. The One-way ANOVA with Tukey’s multiple comparison test is applied in comparisons for groups more than two. Pearson’s rank correlation coefficient was used to assess the correlation between variables in this study. A *P*.adjust less than 0.05 was considered statistically significant.

## Results

### Screening the signature genes in multiple myeloma (MM) by machine learning

**Fig. 1:**
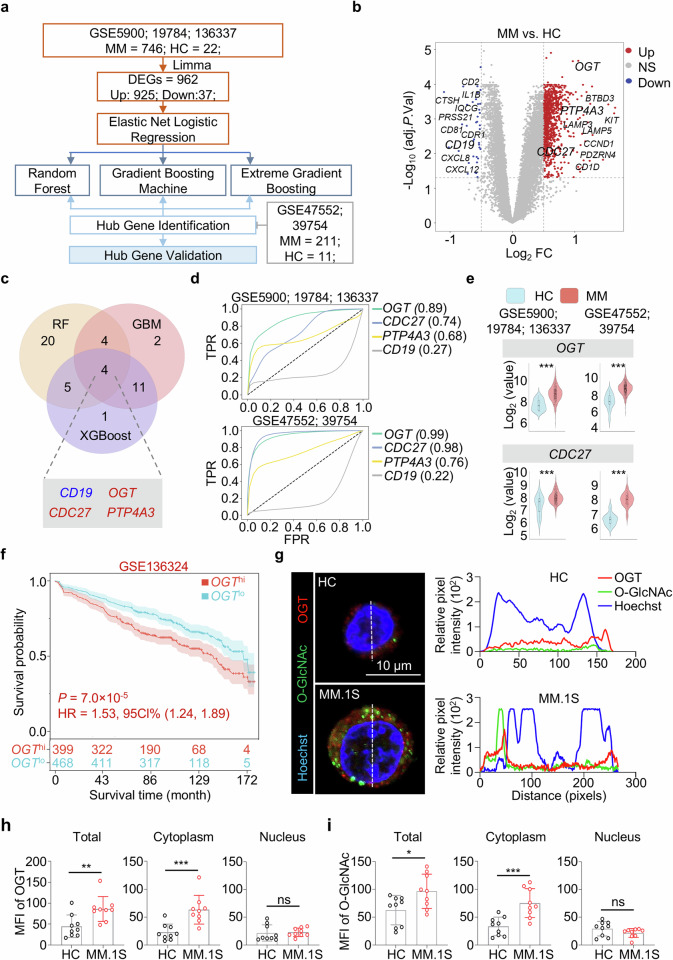
Screening and identification of *OGT* and *CDC27* as key hub genes in MM. **a** Flowchart shows the machine learning strategy. **b** Volcano plot shows the differentially expressed genes between healthy controls (HC) and multiple myeloma (MM) patients. **c** Venn diagram shows the common hub genes from three machine learning models. **d** ROC curves show the hub genes in training set (upper) and testing set (lower). **e** Violin plots show the expression levels of *OGT* and *CDC27* genes in HC and MM patients. **f** Plot shows survival curves in MM patients with high or low *OGT* gene expression levels. **g** Representative images and plots show the localization of OGT (red) and O-GlcNAcylation (O-GlcNAc, green) in HC CD138^+^ plasma cells and MM.1S cells. Scale bar = 10 μm. **h**, **i** Bar charts show the MFI values of OGT and O-GlcNAc in the total, cytoplasmic, and nuclear regions of HC and MM cells. Data were obtained from two independent experiments in **g**–**i**. Data are presented as the means ± SD. Two-tailed Mann‒Whitney U test in **e**. Unpaired two-tailed Student’s *t* test in **h,** **i**. ns: not significant. *P < 0.05, **P < 0.01, ***P < 0.001.

We analyzed multiple previously published datasets to train and validate a diagnostic model for MM. We performed the RNA sequencing analysis of purified myeloma plasma cells samples from five independent MM cohorts (GSE5900; GSE19784; GSE136337; GSE47552; GSE39754). In total, we analyzed the RNA sequencing data from 33 healthy controls (HC) and 957 MM patients with detailed information in Supplementary Table S1. Notably, the GSE5900, GSE19784, and GSE136337 datasets were selected as the primary training dataset for our analysis with gene expression profiles from 746 MM patients and 22 HC. The flowchart of bioinformatic analysis strategy in this study is shown in Fig. 1a.

For identification and enrichment analysis of differentially expressed genes (DEGs), we used the *limma R* package to conduct differential gene analysis and selected genes on the basis of the criteria of a *P*. adjust < 0.05 and a | Log2-Fold Change (FC)| ≥ 0.5. In total, 925 upregulated genes and 37 downregulated genes were identified through a volcano plot via the *ggplot 2* package (Fig. 1b). GO analysis of DEGs showed that the main pathways in biological processes including cytoplasmic translation, ribonucleoprotein complex biogenesis and leukocyte migration (Supplementary Fig. S1a). KEGG analysis suggested that proteasome, protein processing in endoplasmic reticulum and oxidative phosphorylation pathways played important roles in MM pathogenesis (Supplementary Fig. S1b).

Then, the elastic network logistic regression method was employed to identify the feature genes using the *glmnet* package. In total, 87 genes were ultimately identified as input features for machine learning selected with the highest area under the curve (AUC) value obtained from the receiver operating characteristic (ROC) curve of the elastic network logistic regression (Supplementary Fig. S1c). All the gene symbols and their corresponding *P*.adjust values were described in detail in Supplementary Tables S3 and S4.

To screen candidate diagnostic biomarkers of MM, the Gradient boosting machine (GBM), Random forest, and XGBoost algorithms were performed. The GBM offers advantages in terms of high predictive accuracy, overfitting ability, and flexibility. The RF model offers high predictive performance, robustness to overfitting, and feature importance. XGBoost offers advantages in efficiency and speed, high predictive power, and regularization [18]. The performance of each model was evaluated via the ROC curve, and the corresponding AUC value was calculated. All AUC values for the random forest, GBM and XGBoost analysis were higher than 0.98 (Supplementary Fig. S1d).

Notably, 33 genes were identified from the DEGs by using the Random forest algorithm as diagnostic markers. The GBM and XGBoost algorithms were used to identify 21 DEGs as diagnostic markers for MM, respectively. The *CD19*, O-GlcNAc transferase (*OGT*), Cell Division Cycle 27 (*CDC27*), Protein Tyrosine Phosphatase 4A3 (*PTP4A3*) were finally obtained from the overlap of the random forest, GBM and XGBoost analysis (Fig. 1c).

In addition, the expression pattern of four hub genes in common was then validated on independent training (GSE5900, GSE19784, and GSE136337) and test sets (GSE39754 and GSE47552). The results from the ROC analysis revealed that both the *OGT* and *CDC27* genes showed better predictive ability in the training set (AUC = 0.89 and 0.74) and the test set (AUC = 0.99 and 0.98) than the other two hub genes (Fig. 1d). Accordingly, MM patients presented approximately 1.5- to three-fold increase in the expression levels of *OGT* and *CDC27* in both the training set and the test set in MM patients when compared with the healthy individuals, respectively (Fig. 1e). We then validated the survival probability of MM patients with differential *OGT* expression level using the GSE136324 and GSE4204 datasets. The results revealed that MM patients with higher expression levels of *OGT* (*OGT*^hi^) presented lower survival rates and poor prognosis (Fig. 1f and Supplementary Fig. S2).

OGT positively regulates the O-GlcNAcylation of target proteins [19]. To provide a comprehensive understanding of the expression and localization of OGT and O-GlcNAcylation in MM, we compared the expression patterns of OGT and O-GlcNAcylation in purified CD138^+^ plasma cells isolated from healthy control (HC) peripheral blood mononuclear cells (PBMCs) and the MM patient-derived cell line (MM.1S). Immunofluorescence staining revealed extensive accumulated colocalization of OGT and O-GlcNAcylation in the cytoplasmic region of MM.1S cells than HC plasma cells (Fig. 1g). Importantly, further validation of the mean fluorescence intensity (MFI) values revealed an approximately one-fold increase in the total and cytoplasmic OGT and O-GlcNAcylation MFI values from the MM.1S cells. However, no significant changes in nuclear OGT or O-GlcNAcylation levels were detected between control plasma cells and MM.1S cells (Fig. 1h, i).

### O-GlcNAcylation modulates the CDC27 protein expression and suppresses MM cell survival

**Fig. 2:**
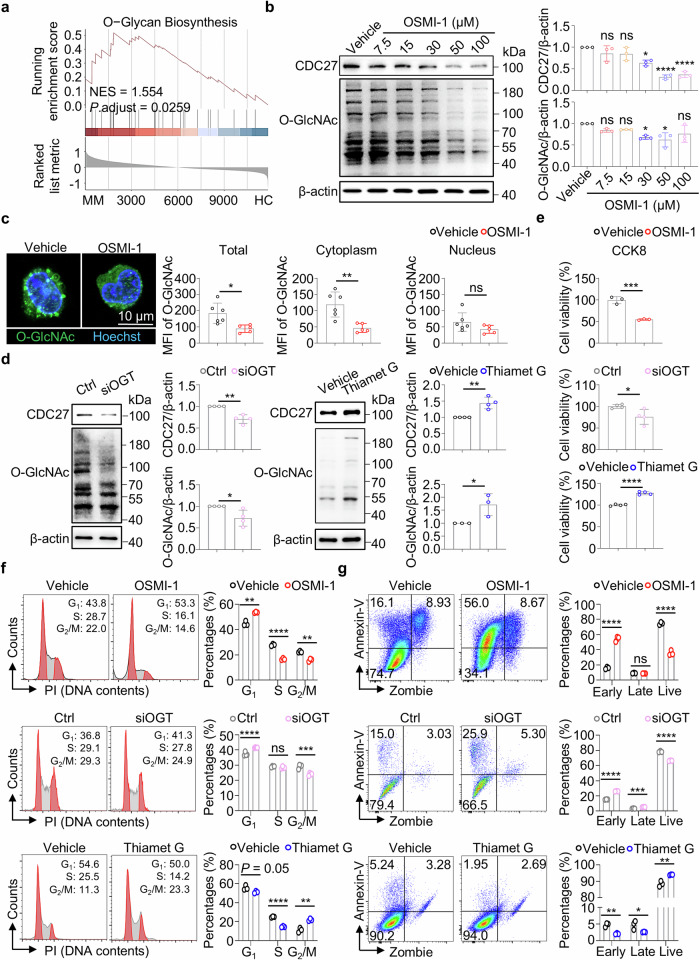
O-GlcNAcylation controls CDC27 expression and cell survival in MM. **a** Gene Set Enrichment Analysis (GSEA) shows the significant enrichment of the O−Glycan Biosynthesis pathway in MM patients. **b** Images and bar charts show the protein levels of CDC27 and O-GlcNAcylation in MM.1S cells treated with the indicated doses of OSMI-1 for 24 h. **c** Representative images and bar charts show the immunofluorescence staining of cytoplasmic, and nuclear O-GlcNAc (green) in MM.1S cells treated with 30 μM OSMI-1 for 24 h. Scale bar = 10 μm. **d** Images and bar charts show the protein levels of CDC27 and O-GlcNAc in MM.1S transfected with 30 nM siOGT for 48 h (left) or treated with 50 nM Thiamet G for 24 h (right). **e** Bar charts show the viability of MM.1S cells treated with OSMI-1 (upper), siOGT (middle), and Thiamet G (lower). Representative flow images and bar charts show the cell cycle analysis **f** and apoptosis analysis **g** of MM.1S cells treated with OSMI-1 (upper), siOGT (middle), and Thiamet G (lower), respectively. All data were obtained from three independent experiments. The data are presented as the means ± SD. One-way ANOVA with Tukey’s multiple comparison test was used in **b**. Unpaired two-tailed Student’s *t* test was used in **c**–**g**. ns: not significant. *P < 0.05, **P < 0.01, ***P < 0.001, ****P < 0.0001.

Using the Gene set enrichment analysis, we identified the significant accumulation of O-Glycan biosynthesis at the top of the ranked gene lists in MM patients (NES = 1.554, *P*.adjust = 0.0259, Fig. 2a). These findings indicated the importance of OGT and O-GlcNAcylation in the pathogenesis of MM.

Then, we used OSMI-1, a selective inhibitor of OGT, to elucidate the role of O-GlcNAcylation in MM (Supplementary Fig. S3a) [20]. We detected the effects of OSMI-1 by the dose- and time-dependent experiments in MM cells. The results revealed a significant downregulation of CDC27 and O-GlcNAcylation protein levels with 30 μM and 50 μM OSMI-1 treatment (Fig. 2b). We then detected kinetic changes of CDC27 and O-GlcNAcylation protein expression levels in MM cells with 30 μM OSMI-1 treatment. Moreover, significant downregulation of CDC27 and O-GlcNAcylation protein levels with OSMI-1 treatment for 24 h was observed (Supplementary Fig. S3b). Interestingly, comparable levels of OGT protein were detected within the dose- and time-dependent experiments (Supplementary Fig. S3b and c), indicating that OSMI-1 mainly inhibited the enzyme activity but not the protein expression in OGT. Then, initial verification by confocal microscopy confirmed a marked decrease in overall O-GlcNAcylation levels, mainly in the cytoplasmic components of MM.1S cells (Fig. 2c). In summary, OSMI-1 effectively suppresses O-GlcNAcylation levels in MM cells.

Next, we analyzed the cellular features of O-GlcNAcylation modification in MM cells with pharmacological inhibition (OSMI-1) and accumulation (Thiamet G) of O-GlcNAcylation, and the knockdown assay of *OGT* gene, respectively [20, 21]. Notably, Thiamet G is a selective inhibitor of O-GlcNAcase (OGA) to pharmacologically accumulate protein O-GlcNAcylation. Firstly, we identified the efficacy of OGT siRNAs (siOGT #1, #2, and #3) by Western blotting. The scrambled siRNA sequence was used as the negative control (Ctrl). As shown in Supplementary Fig. S4, the #2 siOGT showed the best knockdown efficacy with approximately 70% downregulation of OGT protein expression levels. Next, we used the #2 siOGT for functional analysis. Analysis by Western blotting showed the significant downregulation of CDC27 and O-GlcNAcylation protein in MM cells with gene knockdown of *OGT*. Consistently, the upregulation of CDC27 and O-GlcNAcylation protein levels was observed in Thiamet G-treated MM cells (Fig. 2d).

For the cell viability assay, we observed significant decreased MM cell viability in OSMI-1 or siOGT-treated MM cells, while increased MM cell viability was detected in Thiamet G-treated MM cells (Fig. 2e). Analysis of the cell cycle by flow cytometry revealed that MM cell cycle was arrested at the G_1_ phase in OSMI-1-treated or *OGT* knockdown MM cells, while the G_2_/M phase accumulation was detected in Thiamet G-treated MM cells (Fig. 2f). Moreover, subsequent flow cytometry analysis by staining with Annexin V and Zombie Aqua™ Live/Dead Dye revealed the dramatic increase in the early apoptotic stage in OSMI-1-treated or *OGT* knockdown MM cells, while the decreased apoptotic cells were detected in Thiamet G-treated MM cells (Fig. 2g). Here, we show the importance of O-GlcNAcylation in maintaining the viability and division of MM cells.

### CDC27 is the key target of O-GlcNAcylation in MM cells

**Fig. 3:**
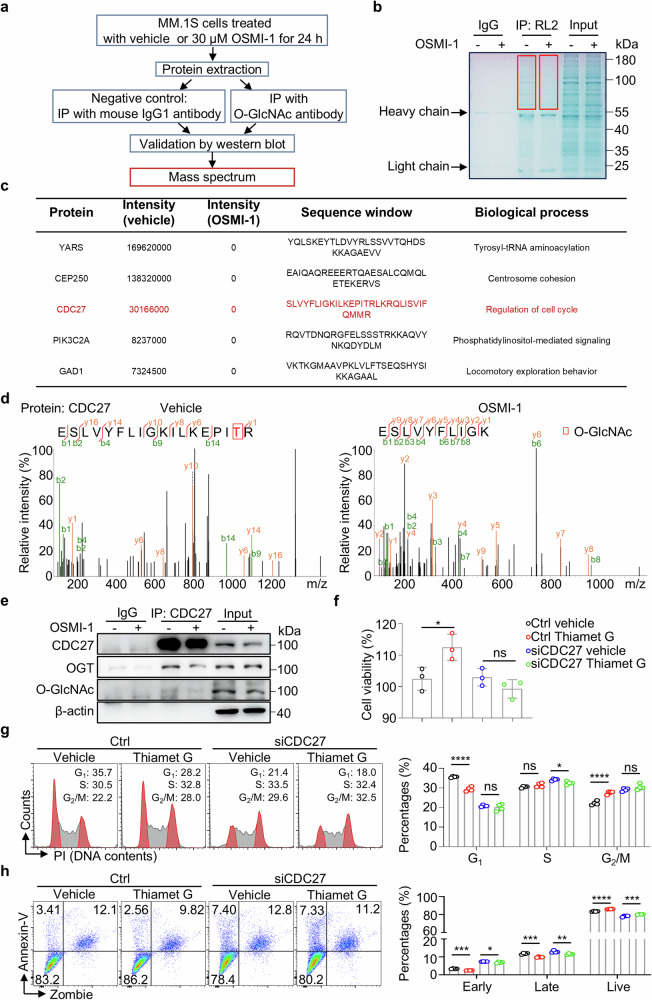
Protein O-GlcNAcylation of CDC27 is inhibited in OSMI-1-treated MM cells. **a** Flowchart shows the strategy of immunoprecipitation (IP) and LC-MS/MS analysis. **b** Coomassie blue staining verifies the protein ladders in IgG control, RL2-precipitated lysis, and input samples. The red box indicates the sample used for LC-MS/MS analysis. **c** Table shows the downregulated peptides and their related functions according to the LC-MS/MS analysis. **d** Plots show the secondary mass spectrum analysis of the CDC27 peptide in vehicle- or OSMI-1-treated MM.1S cells. **e** Representative images showing the binding of CDC27 with OGT, O-GlcNAcylation in vehicle- or OSMI-1-treated MM.1S cells by the Co-IP assay. **f** Bar chart shows the viability of MM.1S cells treated with Ctrl or siCDC27 in the absence or presence of Thiamet G. Representative flow images and bar charts show the cell cycle analysis **g** and apoptosis analysis **h** in MM.1S cells treated with Ctrl or siCDC27 in the absence or presence of Thiamet G by flow cytometry. Data are obtained from two independent experiments in **e** and three independent experiments in **f**–**h**. The data are presented as the means ± SD. One-way ANOVA with Tukey’s multiple comparison test was used in **f**–**h**. ns: not significant. *P < 0.05, **P < 0.01, ***P < 0.001, ****P < 0.0001.

To screen the key targets and underlying mechanisms of O-GlcNAcylation in MM cells, we employed an experimental strategy combining immunoprecipitation with liquid chromatography-tandem mass spectrometry (LC-MS/MS) using MM.1S cells with or without OSMI-1 treatment, as shown in Fig. 3a. The original LC-MS/MS data were uploaded to Supplementary Table S5. Briefly, we collected the whole-cell lysates and immunoprecipitated (IP) with either the anti-O-GlcNAcylation antibody (RL2) or the isotype-matched control IgG. Coomassie blue staining revealed comparable amounts of whole lysis but markedly reduced amounts of RL2 precipitates in MM lysates with the OSMI-1 treatment. Due to the weak bands at 20–25 kDa and 50–55 kDa in IgG control, we then collected gels in the red box (55–180 kDa) for further LC-MS/MS analysis (Fig. 3b). The LC-MS/MS analysis revealed a decrease in protein fragments and peak values following OSMI-1 treatment compared with the vehicle control, indicating successful binding of OSMI-1 to the specific target proteins according to the base peak intensity map analysis (Supplementary Fig. S5). We then identified 5 potential proteins downregulated by OSMI-1 treatment, including YARS, CEP250, CDC27, PIK3C2A, and GAD1 (Fig. 3c). Among these proteins, CDC27 was also identified by the machine learning (Fig. 1) and critically controlled by the O-GlcNAcylation (Fig. 2). Subsequently, we validated CDC27 O-GlcNAcylation by LC-MS/MS analysis and immunoprecipitation. Extraction analysis of CDC27 revealed a reduced O-GlcNAcylation site after treatment with OSMI-1 (Fig. 3d). Here, the LC-MS/MS analysis indicated the direct protein O-GlcNAcylation in CDC27 which could be downregulated by OSMI-1 in MM cells. Further Co-IP assay with the anti-CDC27 antibody confirmed the binding of CDC27 and OGT protein, and the O-GlcNAcylation of CDC27. Moreover, the OSMI-1 treatment markedly reduced CDC27 and OGT binding, as well as the O-GlcNAcylation level of CDC27 (Fig. 3e).

To evaluate the contribution of CDC27 protein O-GlcNAcylation in modulating MM cell function, we used Thiamet G in control or CDC27 knockdown MM cells. Firstly, we identified the efficacy of CDC27 siRNAs (siCDC27 #1, #2, and #3) by Western blotting. The scrambled siRNA sequence was used as control (Ctrl). As shown in Supplementary Fig. S6, the #1 siCDC27 showed the best knockdown efficacy with approximately 90% reduction of CDC27 protein levels when compared with controls. Thus, the #1 siCDC27 was used for further functional analysis.

We analyzed the cell viability, cell cycle, and cell apoptosis via the CCK8 assay and flow cytometry (Fig. 3f–h). As expected, the Thiamet G increased cell viability, which was almost totally blocked by the knockdown of CDC27. Moreover, the Thiamet G-induced G_2_/M phase accumulation and cell apoptosis inhibition were also partially reversed by knockdown of CDC27 in MM cells. Additionally, the knockdown of CDC27 showed no significant changes in the protein levels of O-GlcNAcylated protein in the whole cell lysis (Supplementary Fig. S7). Together, we identified the O-GlcNAcylated CDC27 protein as a key target protein that may be directly downregulated by OSMI-1 in MM.1S cells.

### OSMI-1 promotes CDC27 protein degradation via the autophagy-lysosome pathway in MM cells

**Fig. 4:**
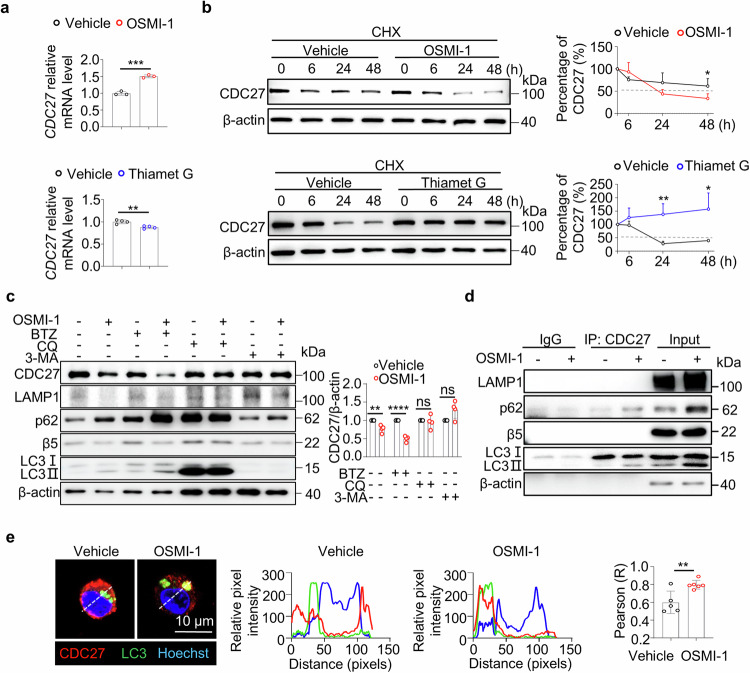
O-GlcNAcylation maintains the CDC27 protein stability through the autophagy-lysosome pathway (ALP) in MM cells. **a** qPCR analysis of *CDC27* mRNA levels in MM.1S cells treated with OSMI-1 (upper) or with Thiamet G (lower). **b** Representative images and plots show the half-life of the CDC27 protein in OSMI-1-treated (upper) or Thiamet G-treated (lower) MM.1S cells incubated with cycloheximide (CHX) for the indicated durations. **c** Images and statistical analysis show the protein levels of CDC27, LAMP1, p62, β5, LC3 in MM.1S cells treated with or without bortezomib (BTZ), 3-methylamphetamine (3-MA), or chloroquine (CQ) in the absence or presence of OSMI-1 for 24 h. **d** Representative images show the binding between CDC27 and LAMP1, p62, β5, and LC3II/I in OSMI-1-treated MM.1S cells *via* the Co-IP assay. **e** Representative images and plots show the colocalization of CDC27 (red) and LC3 (green) in OSMI-1-treated MM.1S cells by confocal microscopy. Scale bar = 10 μm. The data were obtained from three independent experiments. The data are presented as the means ± SD. Unpaired two-tailed Student’s *t*-test was used in **a,** **b, c**, and **e**. ns: not significant. *P < 0.05, **P < 0.01, ***P < 0.001, ****P < 0.0001.

To elucidate the underlying mechanism of CDC27 O-GlcNAcylation in modulating MM cell function, firstly, we observed the increased *CDC27* mRNA levels in OSMI-1-treated MM cells but reduced levels in Thiamet G MM cells, which is contrary to the protein level changes in MM cells (Fig. 4a), indicating the protein levels of CDC27 were crucially modulated at the post-transcriptional levels.

To further investigate the role of O-GlcNAcylation in controlling the CDC27 protein stability, we preincubated cells with an inhibitor of eukaryotic protein synthesis (cycloheximide, CHX). A significantly reduced half-life of the CDC27 protein was observed in OSMI-1 treated MM cells but increased CDC27 protein stability was indicated in Thiamet G-treated MM cells (Fig. 4b). These findings indicate that O-GlcNAcylation maintained the protein stability of CDC27 in MM cells.

Two main signaling pathways are involved in the protein degradation, including the ubiquitin-mediated proteasome pathway and the autophagy-lysosome pathway [18, 22]. To explore the pathways related to the acceleration of CDC27 protein degradation by OSMI-1 treatment, we then detected the levels of proteins related to two major protein degradation pathways, β5 (a proteasome protein), ubiquitin, p62 (an autophagy-related protein), LC3 II/I (an autophagy protein), and LAMP1. The representative Western blotting images and statistical analysis revealed elevated protein levels of p62 and LC3 II/I but comparable or decreased levels of β5, ubiquitin, and LAMP1 in OSMI-1-treated MM.1S cells (Supplementary Fig. S8a). Then, we incubated MM cells with BTZ (a proteasome inhibitor), 3-MA (an autophagy inhibitor), or CQ (a late autophagy and lysosome inhibitor) to block the related pathways involved in protein degradation, separately. Our results showed that the downregulation of CDC27 protein levels by OSMI-1 treatment was significantly reversed in the presence of 3-MA or CQ. However, BTZ could not block OSMI-1-induced CDC27 protein degradation (Fig. 4c). The efficacy of BTZ, 3-MA, and CQ on the protein levels of β5, p62, LC3 II/I, and LAMP1 was shown in Fig. 4c and Supplementary Fig. S8b. Our results suggest the crucial involvement of the autophagy-lysosome pathway (ALP) in OSMI-1-induced CDC27 protein degradation.

The envelopment of the autophagy-lysosome pathway (ALP) in OSMI-1-induced CDC27 protein degradation was also validated by the co-IP assay. The enhanced binding of CDC27 with p62 and LC3 II was observed in OSMI-1-treated MM.1S cells. However, the proteasome subunit β5 could not be detected by immunoprecipitation with the anti-CDC27 antibody (Fig. 4d). We also confirmed the significant colocalization of cytoplasmic CDC27 and LC3 in OSMI-1-treated MM.1S cells by confocal microscopy (Fig. 4e).

Moreover, a significant increase in the MFI values of the lysosome signal with LysoTracker staining after 24 h of OSMI-1 treatment suggested that OSMI-1 increased the degradation capacity (Supplementary Fig. S9a). Additionally, the colocalization of CDC27 and LAMP1 was detected in OSMI-1-treated MM.1S cells, suggesting lysosome-dependent CDC27 protein degradation by OSMI-1 treatment (Supplementary Fig. S9b). In summary, our findings indicate that ALP-mediated protein degradation of CDC27 is an alternative pathway to OSMI-1 treatment in MM cells.

### OSMI-1 decreases cell viability via the autophagy-lysosome pathway in MM cells

**Fig. 5:**
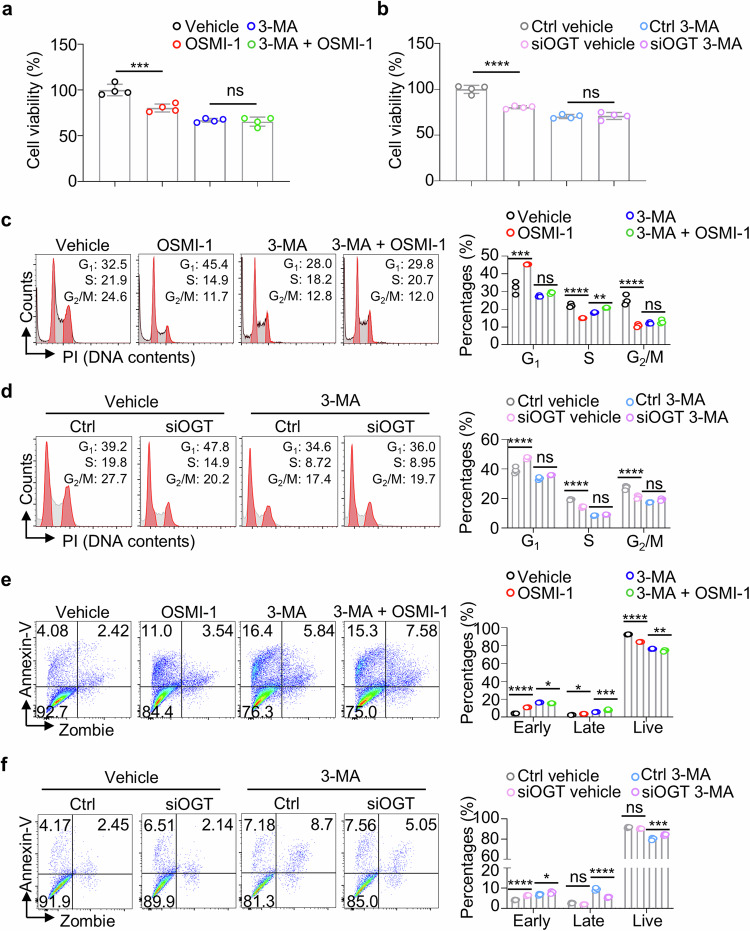
O-GlcNAcylation controls the MM cell function *via* autophagy. Bar charts show the viability of MM.1S cells treated with 3-MA (1 mM) in the absence or presence of OSMI-1 **a** or gene knockdown with siOGT **b**. Representative images and bar charts show the flow cytometry analysis of the cell cycle in MM.1S cells treated with 3-MA in the absence or presence of OSMI-1 **c** or siOGT **d**. Representative images and bar charts show the flow cytometry analysis of cell apoptosis of MM.1S cells treated with 3-MA in the absence or presence of OSMI-1 **e** or siOGT **f**. The data were obtained from three independent experiments. The data are presented as the means ± SD. One-way ANOVA with Tukey’s multiple comparison test was used in **a**–**f**. ns not significant. *P < 0.05, **P < 0.01, ***P < 0.001, ****P < 0.0001.

Since the ALP-dependent CDC27 protein degradation may be directly involved in the survival and proliferation of MM.1S cells, we then validated cell features with the autophagy inhibitor 3-MA in the OSMI-1 treated or *OGT* knockdown MM cells. CCK8 assay revealed that the OSMI-1 or siOGT-induced reduction in cell viability could be almost totally reversed by blocking autophagy with 3-MA (Fig. 5a, b). Moreover, further analysis of the cell cycle by flow cytometry revealed that 3-MA incubation partially abrogated cell cycle arrest at the G_1_ phase caused by the OSMI-1 treatment or knockdown of OGT in MM cells (Fig. 5c, d). Flow cytometry analysis also demonstrated the partial blockade of OSMI-1-or siOGT-induced MM cell apoptosis by the 3-MA treatment in MM cells (Fig. 5e, f). Here, we highlight the importance of ALP-dependent CDC27 protein degradation by OSMI-1 treatment and the role of ALP in maintaining cell viability and the cell cycle in MM cells.

### The synergistic effects of O-GlcNAcylation inhibition and BTZ in MM cells by the combination index (CI) analysis

**Fig. 6:**
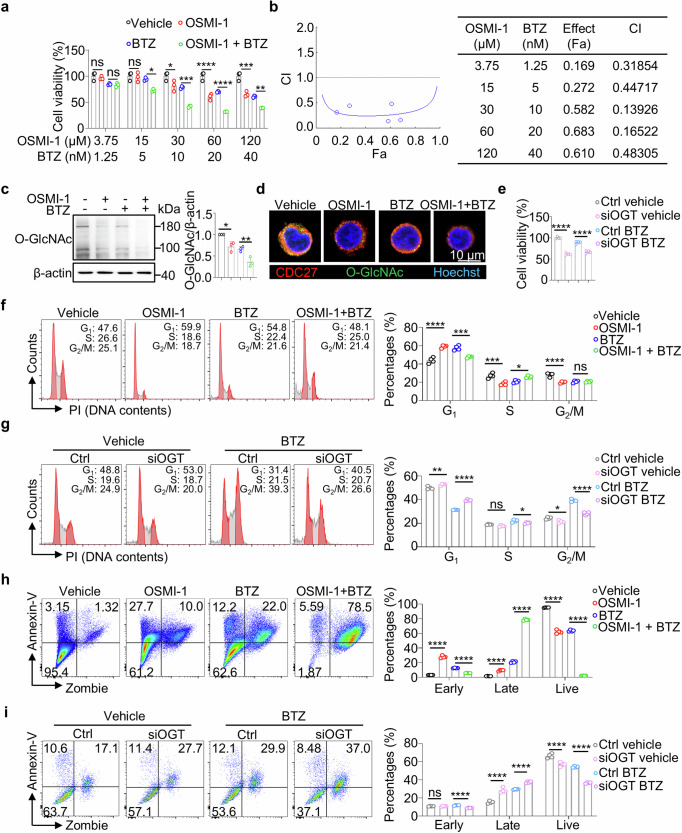
OSMI-1 treatment improves the therapeutic effects of BTZ in MM. **a** Bar chart shows the viability of MM.1S cells treated with or without BTZ and OSMI-1 for 24 h. The combination treatments were performed in MM.1S cells maintaining a constant ratio (3:1) between the dose of the OSMI-1 and BTZ. **b** The Combination Index (CI) was calculated using the CalcuSyn software to analyze the combined effect of OSMI-1 and BTZ. CI < 1 indicates the synergistic effects. **c** Images and statistical analysis show the protein levels of O-GlcNAcylation in MM.1S cells treated with or without BTZ and OSMI-1. **d** Representative images showing the immunofluorescence staining of CDC27 (red) and O-GlcNAcylation (green) in MM.1S cells treated with or without BTZ and OSMI-1. Scale bar = 10 μm. **e** Bar chart shows the viability of MM.1S cells treated with or without BTZ and gene knock down with siOGT. Representative images and bar charts showing the results of flow cytometry analysis of the cell cycle of MM.1S cells treated with or without BTZ and OSMI-1 **f** or siOGT **g**. Representative images and bar charts showing the results of flow cytometry analysis of the cell apoptosis of MM.1S cells treated with or without BTZ and OSMI-1 **h** or siOGT **i**. The data were obtained from three independent experiments. The data are presented as the means ± SD. One-way ANOVA with Tukey’s multiple comparison test was used in **a, c, e**–**i**. ns: not s**i**gnificant. *P < 0.05, **P < 0.01, ***P < 0.001, ****P < 0.0001.

Due to the key role of ALP in the mechanism of O-GlcNAcylation inhibition, OSMI-1 may harbor the potential synergistic effects with the classic MM drug, BTZ (a proteasome inhibitor). To evaluate the synergistic effects of O-GlcNAcylation inhibition and BTZ, we calculated the combination index (CI) using five drug concentrations of OSMI-1 and BTZ. The cell viabilities were assessed with single or combined treatment. Our results showed the Fa values were less than 0.7, indicating the synergistic effects of BTZ and OSMI-1. Moreover, a combination of OSMI-1 (30 µM) and BTZ (10 nM) resulted in the optimal synergistic inhibition, where the CI value was approximately 0.14 (Fig. 6a, b).

For the mechanistic study, we demonstrated the significant decrease in the O-GlcNAcylation levels with combined use of OSMI-1 and BTZ in MM cells (Fig. 6c). Moreover, confocal microscopy confirmed the decreased trend in the colocalization signal of CDC27 and O-GlcNAcylation by the OSMI-1 combined BTZ treatment (Fig. 6d).

To further investigate the synergistic effects of O-GlcNAcylation inhibition and BTZ in MM cells, cell survival and proliferation status were assessed. The OSMI-1 or siOGT treatment was applied to inhibit O-GlcNAcylation in MM cells. Compared with BTZ treatment alone, combination treatment with OGT knockdown significantly decreased the viability of MM cells, as determined by the CCK8 assay (Fig. 6e). Notably, the BTZ treatment reversed OSMI-1 or siOGT-induced G_1_ phase arrest in MM cells (Fig. 6f, g). However, no significant synergistic effects of O-GlcNAcylation inhibition and BTZ were observed in MM cell cycle. Furthermore, the combination treatment of O-GlcNAcylation inhibition and BTZ appeared to dramatically increase late-stage apoptosis and reduce the live cell frequencies in MM cells (Fig. 6h, i).

In addition, Western blotting assay showed comparable protein level of OGT and significant decrease in the protein level of CDC27 and an increase in the ratio of LC3 II/I in MM cells with combined treatment of OSMI-1 and BTZ (Supplementary Fig. S10a and b). These findings indicated the potential synergistic therapeutic efficacy of O-GlcNAcylation inhibition and BTZ in enhancing the apoptotic signaling in MM cells.

### The synergistic effects of OSMI-1 and BTZ in a xenograft model of MM

**Fig. 7:**
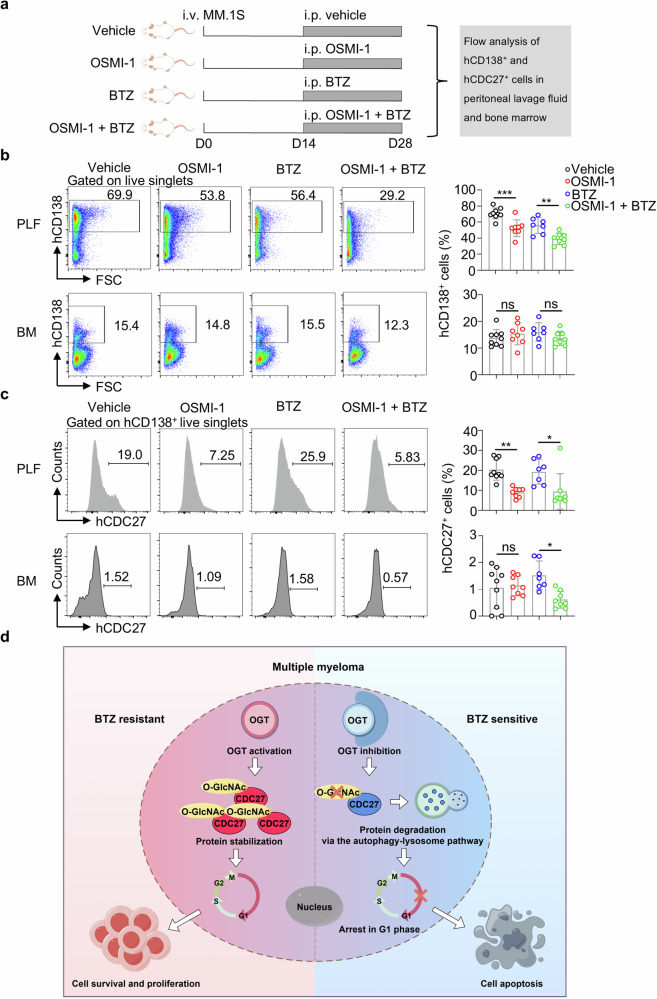
OSMI-1 treatment improved the therapeutic effects of BTZ in vivo. **a** Experimental scheme showing the xenograft model and combined MM treatment in this study (*n* = 9 in the vehicle group, *n* = 8 in the OSMI-1-treated group, *n* = 7 in the BTZ-treated group, and *n* = 8 in the OSMI-1 combined with BTZ-treated group). **b** Representative images and flow cytometry analysis of the percentages of hCD138^+^ MM.1S cells in the peritoneal lavage fluid and bone marrow of recipient NSG mice as indicated. **c** Representative images and flow cytometry analysis of hCDC27^+^ subsets within hCD138^+^ MM.1S cells in the peritoneal lavage fluid and bone marrow of recipient NSG mice are shown as indicated. **d** Diagram shows the research hypothesis of this study. The data are presented as the means ± SD. One-way ANOVA with Tukey’s multiple comparison test was used in **b, c**. ns: not significant. *P < 0.05, **P < 0.01, ***P < 0.001.

BTZ is the frontline chemotherapy drug for MM. Currently, resistance to BTZ poses a significant challenge in the treatment of MM [23]. We aimed to investigate whether a combination strategy with OSMI-1 could enhance the therapeutic efficacy of BTZ in MM. Initially, we established an MM model in immunodeficient NSG mice through tail vein injection of MM.1S cells as reported previously [24]. Two weeks after xenotransplantation, we administered 5 mg/kg OSMI-1 and 0.5 mg/kg BTZ via the intraperitoneal injection (i.p., Fig. 7a).

For quantification of the cellular source in our xenograft model, we first identified surface biomarkers for MM.1S cells. Flow cytometric analysis revealed a positive staining signal for surface CD138 but no surface CD19 or CD27 signal in MM.1S cells when compared to the fluorescence minus one (FMO) control, respectively (Supplementary Fig. S11). Additionally, we detected human CD138-positive cells in the peritoneal cavity and bone marrow of NSG mice with or without MM.1S cell transfer via flow cytometry. Notably, surface human CD138 (hCD138)-positive subsets were detected in transplanted NSG mice with approximately 73% in the peritoneal lavage fluid (PLF) and 20% in the bone marrow (BM, Supplementary Fig. S12). The combined treatment of OSMI-1 and BTZ resulted in the lowest frequencies of hCD138^+^ cell subsets in both the peritoneal cavity and bone marrow of combined OSMI-1- and BTZ-treated recipients compared with the OSMI-1- or BTZ-treated recipients and the vehicle controls (Fig. 7b). Furthermore, we detected the CDC27 signal within hCD138^+^ cell subsets in recipient mice via flow cytometry. The results revealed the lowest frequencies of CDC27^+^ cells within the hCD138^+^ subsets in both the peritoneal cavity and bone marrow of combined OSMI-1- and BTZ-treated recipients (Fig. 7c). Thus, our xenograft model revealed the synergistic effects of OSMI-1 and BTZ in MM in vivo. In conclusion, our findings highlight the importance of O-GlcNAcylation for CDC27 protein stability and the antitumor efficacy of combined OSMI-1 and BTZ treatment (Fig. 7d).

## Discussion

MM is a hematologic malignancy characterized by the abnormal proliferation of cloned plasma cells. Although treatments for MM have improved in recent years, drug resistance is becoming a great challenge in patients with relapsed refractory MM [25]. Drug resistance in MM remains largely unclear. Therefore, the screening and identification of effective and novel diagnostic biomarkers for MM may facilitate the early diagnosis and therapeutic landscape of MM patients. Here, we first applied comprehensive machine learning models, including the gradient boosting machine, random forest and XGBoost models, to screen the hub genes for MM. The gradient boosting machines and extreme gradient boosting optimize the prediction performance by iteratively building models, which are particularly effective in handling nonlinear relationships and interactions among biological data. In addition, random forests improve model stability and accuracy by constructing multiple decision trees and making ensemble predictions [26, 27]. The combination of three machine learning models is crucial for identifying specific biomarkers and can provide deeper insights into the intricate biological processes underlying MM pathogenesis.

In the present study, four signature marker genes (*OGT, CDC27, CD19*, and *PTP4A3*) were identified through the application of gradient boosting machine, random forest and XGBoost analysis. Compared with the other hub genes, the *OGT* gene had greater predictive ability in the training and test sets. The elevated expression of *OGT* is also related to an unfavorable prognosis and lower survival rates in MM patients. These findings indicate that the marker gene *OGT* is crucially involved in the pathogenic process of MM. Moreover, our experimental data suggest that the CDC27 might be one major target for OGT-mediated protein O-GlcNAcylation in MM. In addition, the chimeric antigen receptor (CAR) T Cells against another hub gene *CD19* for the treatment of MM with a complete response with no evidence of progression [28]. Moreover, MM patients with high expression level of *PTP4A3* mRNA showed poor prognosis [29]. The pro-metastatic PTP4A3 maintained MYC expression in t (4;14) multiple myeloma. Together, the machine learning models used in this study appear to effectively identify valuable marker genes for MM.

In MM patients, the overexpression of OGT is directly linked to O-GlcNAcylation and disease progression, indicating that inhibiting O-GlcNAcylation may be a promising strategy for treating MM. O-GlcNAcylation is a dynamic and reversible protein posttranslational modification that contributes to numerous cellular functions, including signaling, protein localization and stability, transcription, chromatin remodeling, mitochondrial function, and cell survival [30, 31]. The inhibition of O-GlcNAcylation has also been suggested to decrease chemoresistance in ovarian cancer, breast cancer, and many metabolic disorders [32, 33]. In this study, increased O-GlcNAcylation in the cytoplasmic region of MM cells may directly influence the growth, survival, and drug resistance of MM cells.

OSMI-1 is a novel, small cell-permeable molecule that inhibits OGT [34]. Previous studies have reported that OSMI-1 can modulate the function of tumor cells [35, 36]. Overall, we reported that OSMI-1 inhibits cell proliferation and promotes cell apoptosis by blocking the cell cycle at the G_1_ phase in MM cells. The effects of OSMI-1 were also confirmed using the gene knockdown of the OGT. In fact, the identification and screening of OGT inhibitors with lower toxicity has led to the development of OGT inhibitors as effective therapeutic targets for several diseases. The crystal structure of OGT with the donor substrate and the target polypeptide substrate has been determined previously, and several OGT inhibitors have been obtained from the modification of the donor substrate UDP–GlcNAc [37]. For instance, the OGT donor substrate mimics and competes with UDP, alloxan, compound 4, BZX and OSMI-1 [34]. As OSMI-1 may influence cell viability, novel OGT inhibitors with lower cytotoxicity are needed for further clinical use.

O-GlcNAcylation modifications can occur on specific amino acids to control protein stability [38]. Here, we identified CDC27 as a target protein for O-GlcNAcylation and a key target protein of OSMI-1 via LC-MS/MS analysis and immunoprecipitation. CDC27 regulates the cell cycle by forming the anaphase-promoting complex (APC/C) and mediating the ubiquitin-mediated proteolysis of cyclins [39]. In addition, CDC27 has been reported to be upregulated in patients with acute myeloid leukemia (AML) or acute lymphoblastic leukemia (ALL) [40]. However, modifications and regulations of CDC27 have rarely been reported in MM.

In our study, we found that OSMI-1 decreased CDC27 protein stability by reducing the O-GlcNAcylation of CDC27. Further studies using site mutations to identify the O-GlcNAcylation site of CDC27 in MM cells are needed. Moreover, the subsequent activation of the ubiquitin-mediated proteasome pathway and the autophagy-lysosome pathway (ALP) might be involved in CDC27 protein degradation [41]. The inhibition of the autophagy and lysosome pathways effectively abrogated the effects of OSMI-1 in MM cells. Previous studies also reported that OSMI-1 induces autophagy in bladder cancer and colon cancer [35, 36]. Here, we reported that OSMI-1 could degrade the CDC27 protein through the ALP during the downregulation of the protein O-GlcNAcylation of CDC27 in hematologic malignancies.

BTZ has been applied as a first-line therapeutic drug in the treatment of MM. Recent studies have shown that BTZ-induced apoptosis is dependent on the accumulation of unfolded proteins in the endoplasmic reticulum (ER)-dependent proapoptotic process in MM cells [25]. Previous studies have revealed that the mitochondrial stress pathway, oxidative stress pathway, and SUMOylation are involved in the disease progression and drug resistance of MM [42–46]. The ubiquitin-proteasome system has been reported to be associated with certain types of selective autophagy [4, 45]. In our study, we discovered that OSMI-1 may trigger the activation of the autophagy-lysosome pathway independent of the ubiquitin-proteasome system.

The resistance of MM to BTZ has gradually emerged and has become a major challenge in the treatment process. Low expressions of XBP1, ATF3, and ATF4, have been identified in poor responders to BTZ treatment [47]. Moreover, the current study used single-cell RNA sequencing to define the specific features of BTZ resistance in MM patients. Moreover, MM-specific NK cells and T cells can also affect BTZ treatment responsiveness via distinct cellular and gene networks [6]. The combination of anticancer drugs and chemical sensitizers is commonly used to increase therapeutic efficacy [25]. In our investigation, we focused on whether the combination of BTZ and OSMI-1 can improve treatment efficacy in MM patients. Our data suggested that the combined treatment of BTZ and OSMI-1 synergistically enhanced anticancer activity in vivo and in vitro. A mechanistic study revealed that combination therapy with BTZ and OSMI-1 further abrogated cell cycle arrest in the G_1_ phase and promoted the apoptosis of MM cells. We observed the upregulation of p62 protein in OSMI-1 treated MM cells, the pharmacologic targeting of the p62 ZZ domain could enhance the anti-tumor and bone-anabolic effects of bortezomib in MM as well [48]. Besides, other novel targets in the treatment of MM have been indicated, such as the humanized B-cell maturation antigen (BCMA)-CD3 bispecific antibody [49]. Moreover, the effective treatment of CAR-T also has been applied in MM study [50]. Whether OSMI-1 has synergistic effects with other therapies in MM treatment still needs further investigation.

In conclusion, we demonstrate that O-GlcNAcylation is involved in the occurrence and poor prognosis of MM. The use of OSMI-1 and gene knockdown of *OGT* to reduce the O-GlcNAcylation of CDC27 can induce cell cycle arrest in the G_1_ phase and cell apoptosis. Furthermore, OSMI-1 notably reduced the protein stability of CDC27 by activating the ALP. In the MM mouse model, combination therapy with OSMI-1 and BTZ resulted in significant inhibition of MM cell growth, with downregulated expression of the CDC27 protein. In addition, we demonstrated the underlying mechanism of the synergistic effect of OSMI-1 and BTZ through alternative autophagy-lysosome pathway-mediated CDC27 protein degradation. Thus, O-GlcNAcylation of CDC27 maintains the drug resistance of BTZ in MM. The combination of OSMI-1 and BTZ may provide a potential strategy for the treatment of refractory MM.

## Supplementary information

Supplementary Figures S1-S12

Supplementary Tables
