# Supplementary figures and images for: Inhibition of CDC27 O-GlcNAcylation coordinates the antitumor efficacy in multiple myeloma through the autophagy-lysosome pathway
Source: Acta Pharmacol Sin. 2025 Feb 21;46(7):2041–55. doi: 10.1038/s41401-025-01500-2 (PMC12205061; doi:10.1038/s41401-025-01500-2)

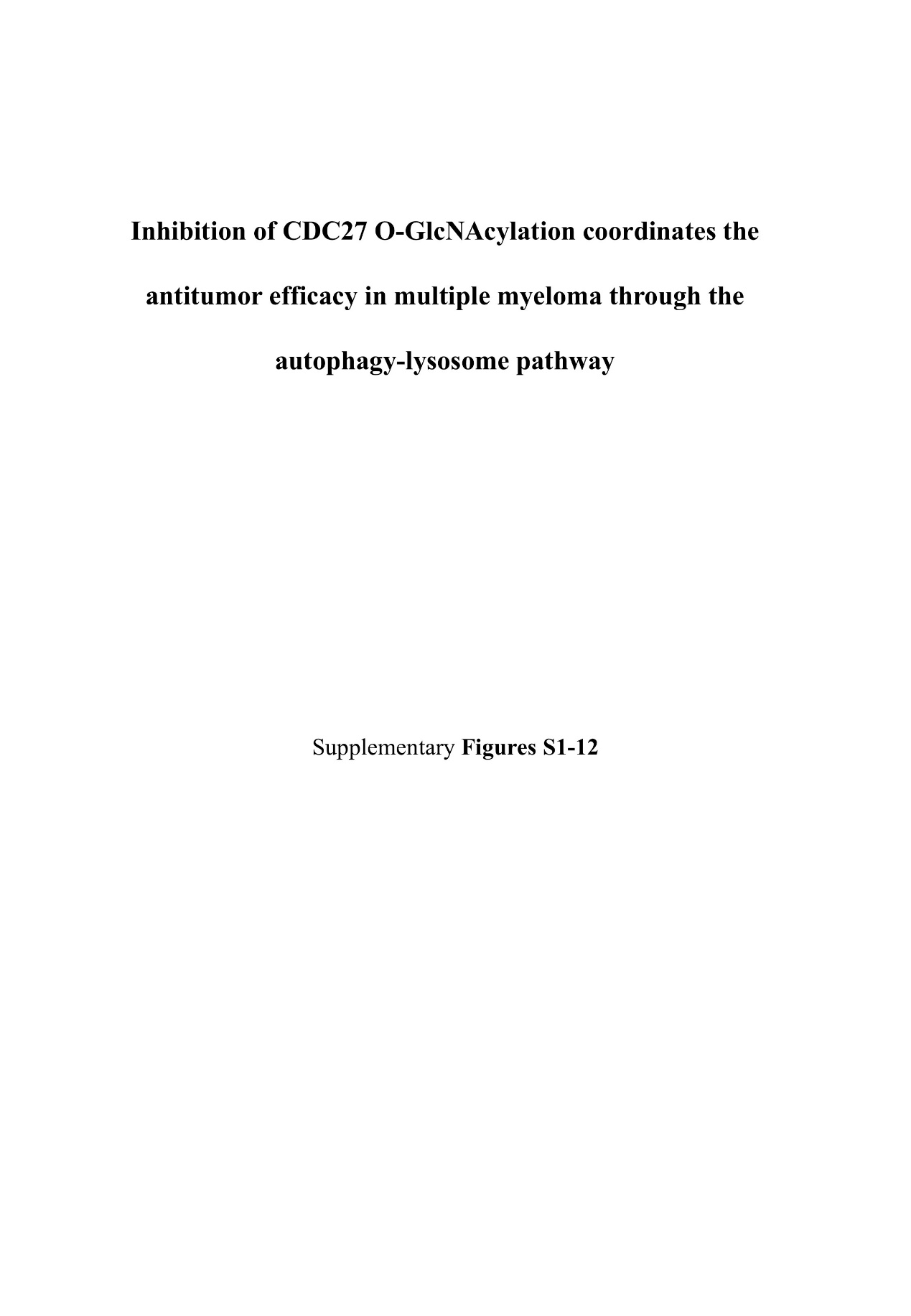


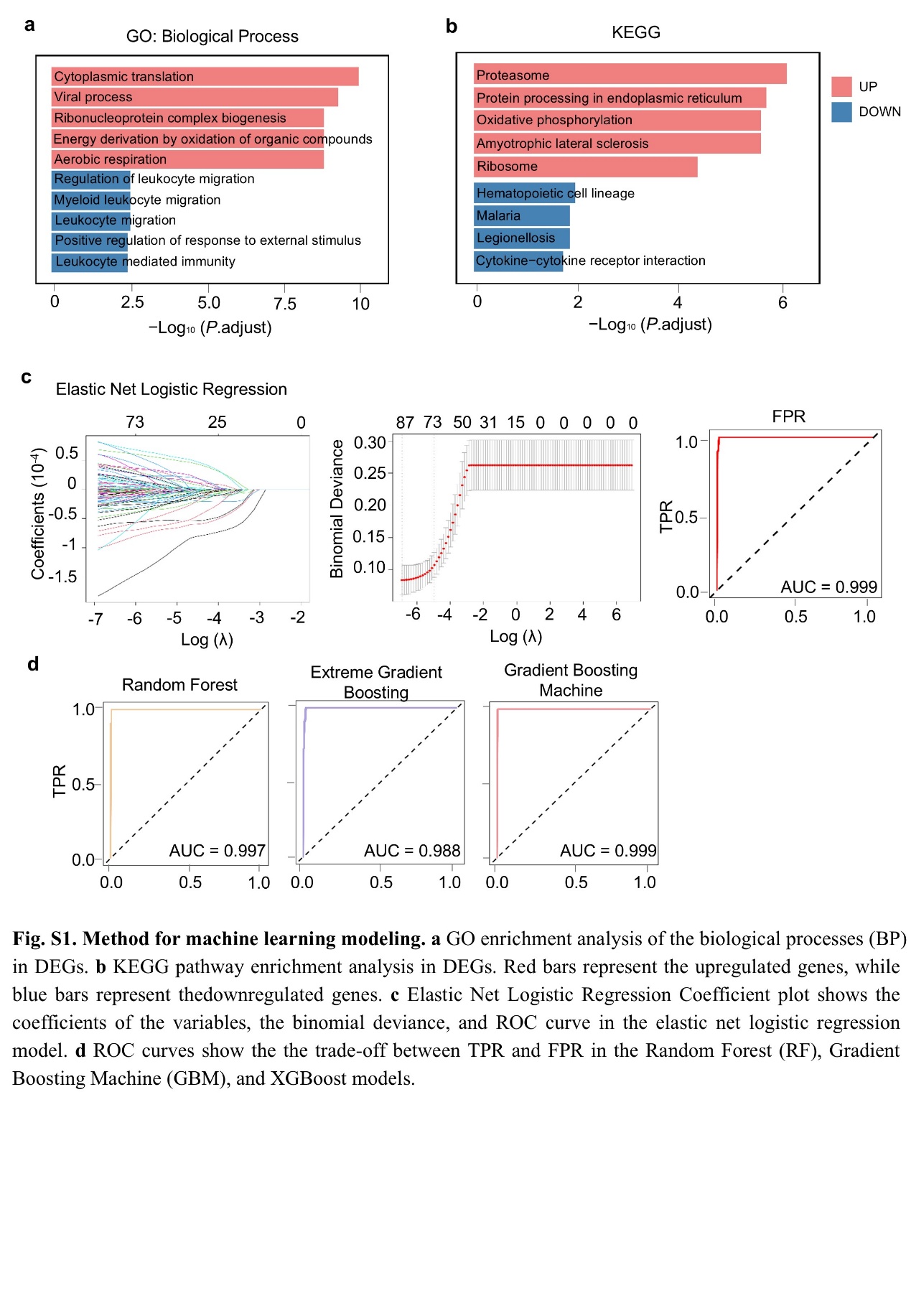


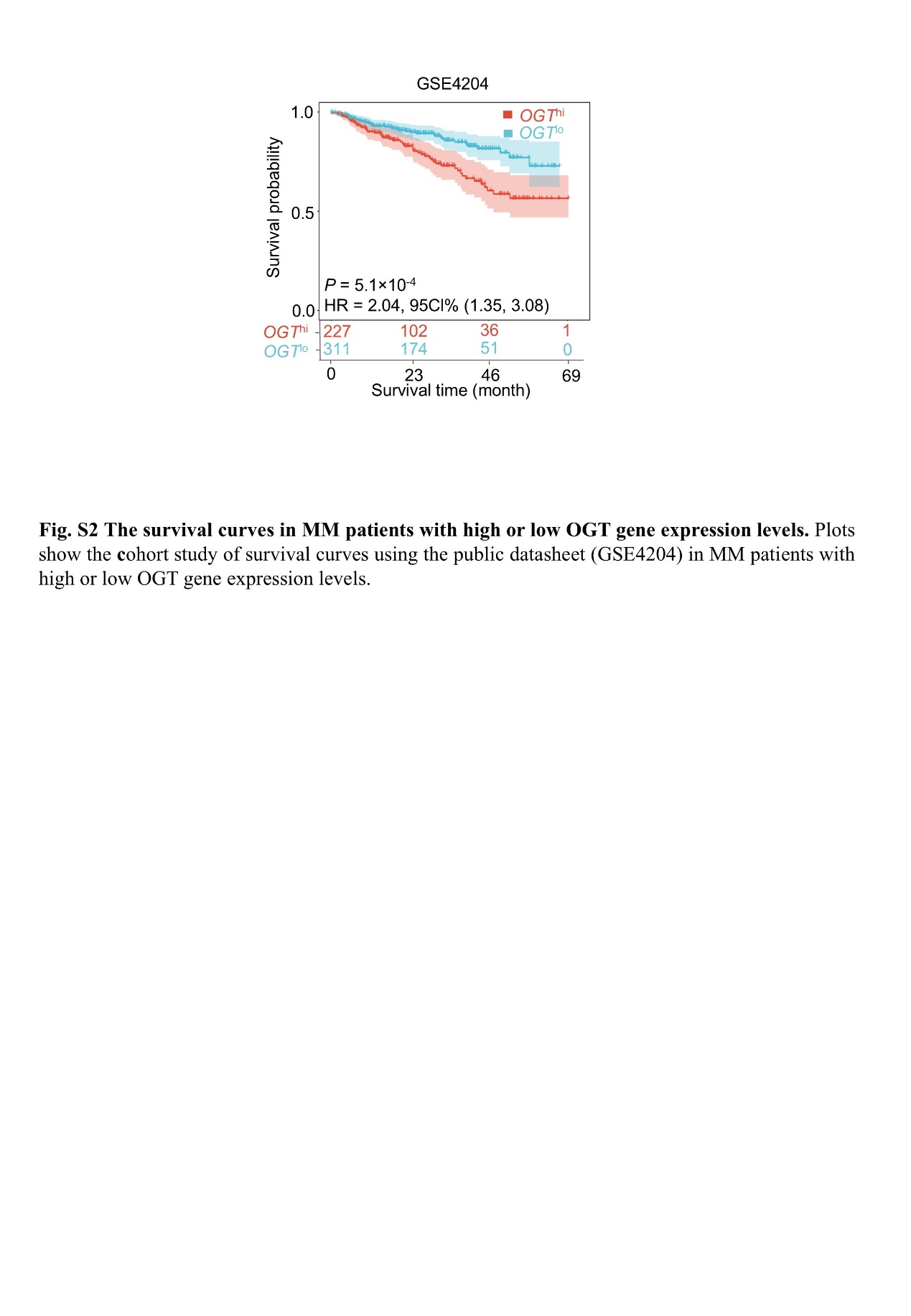


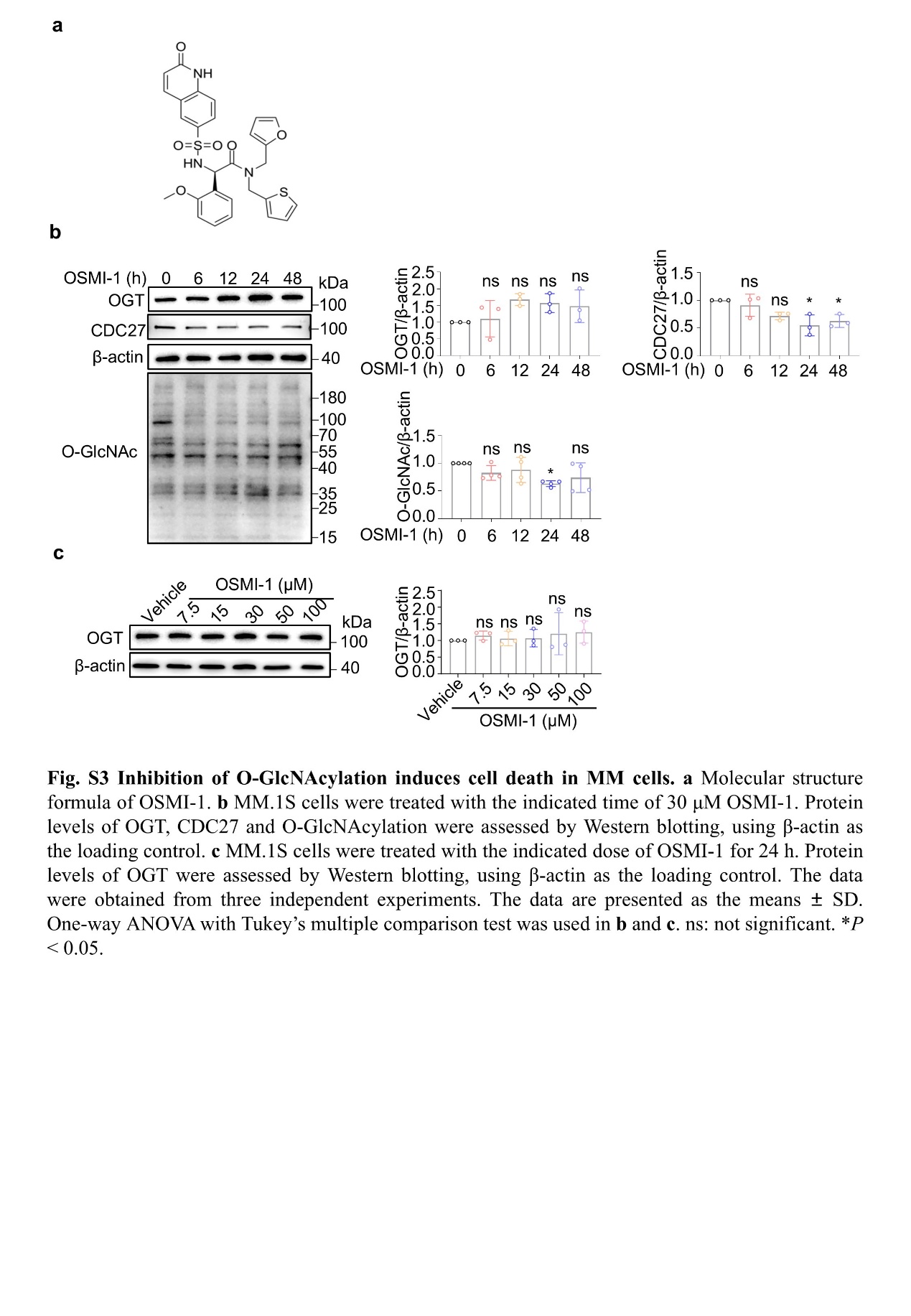


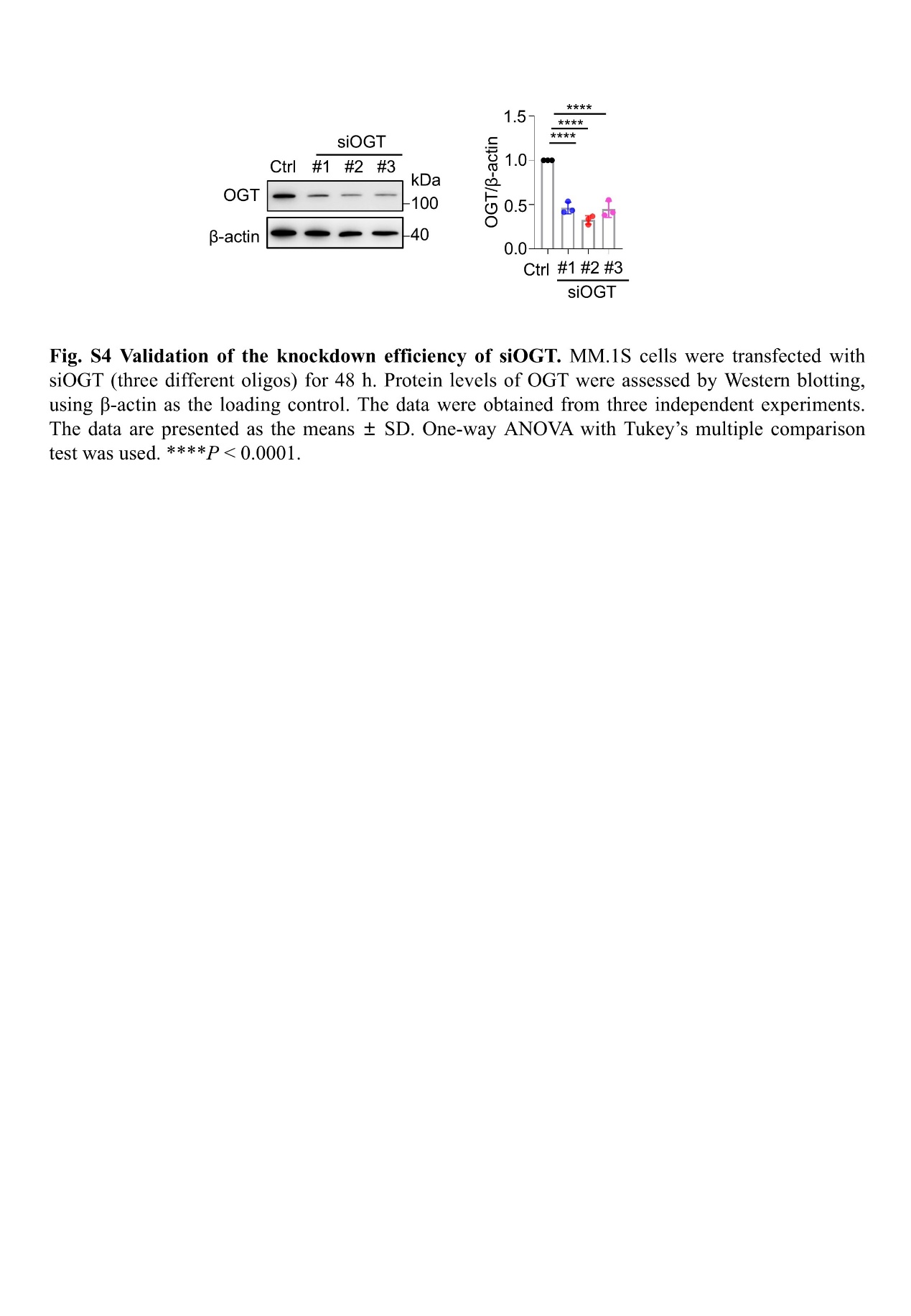


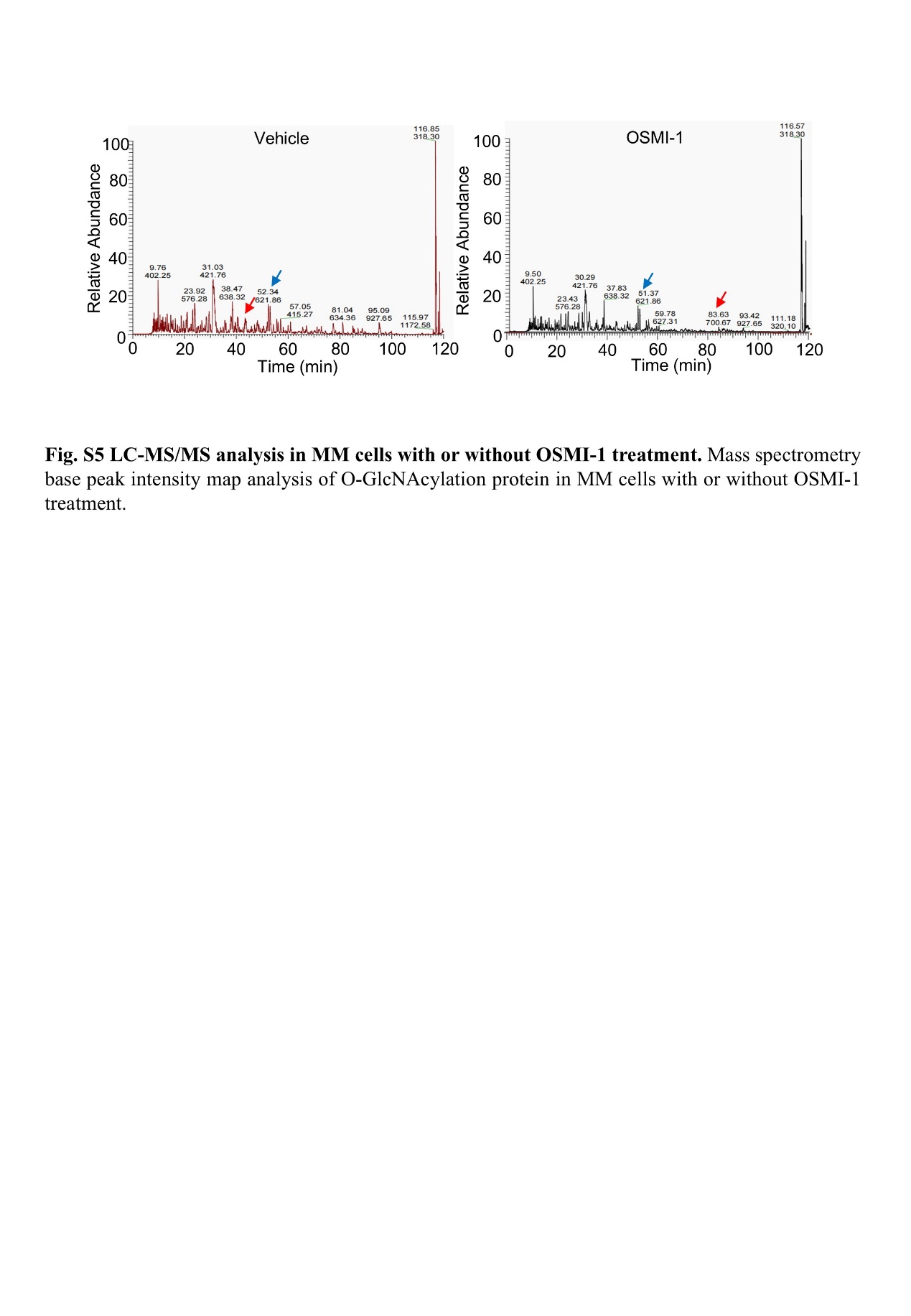


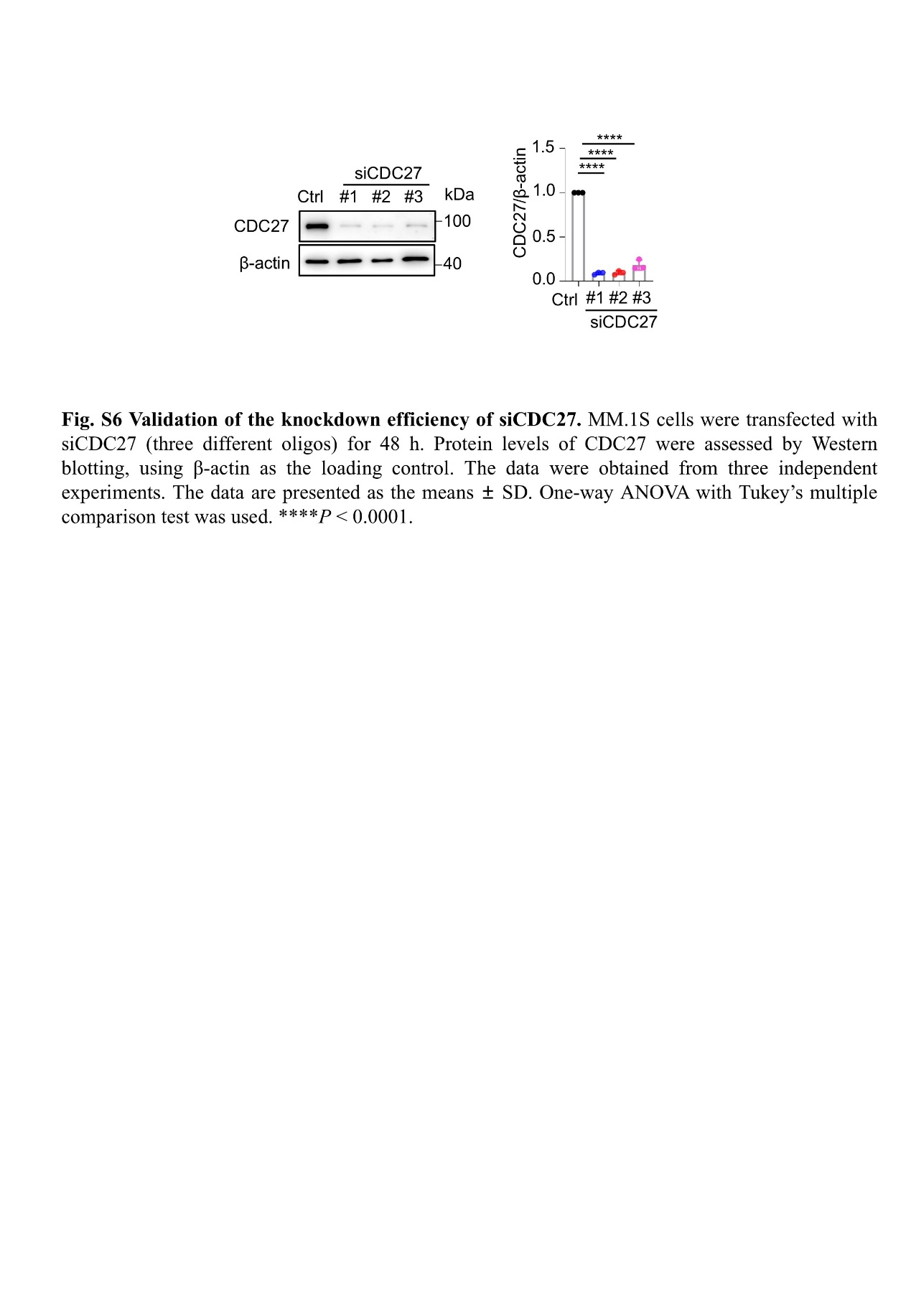


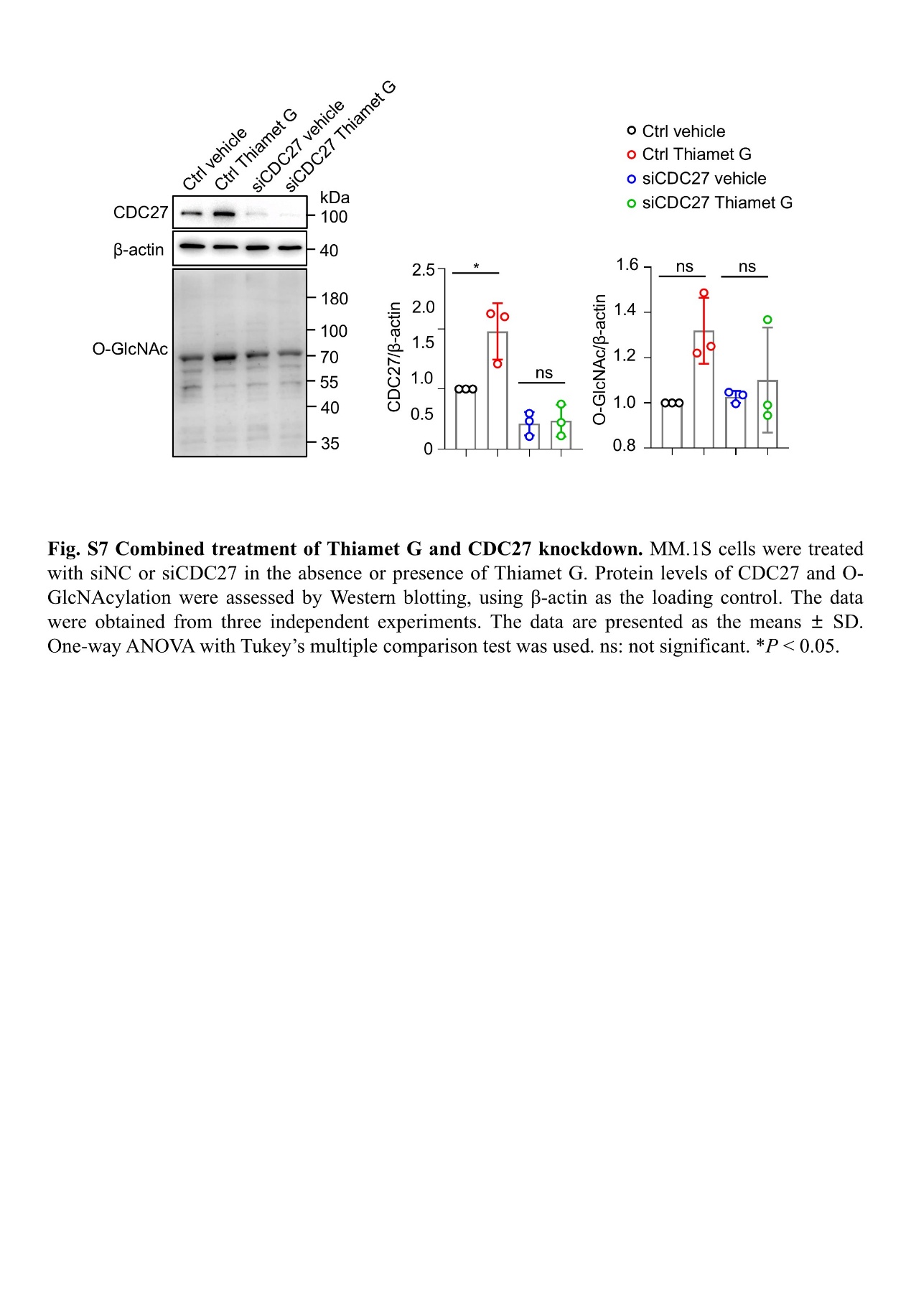


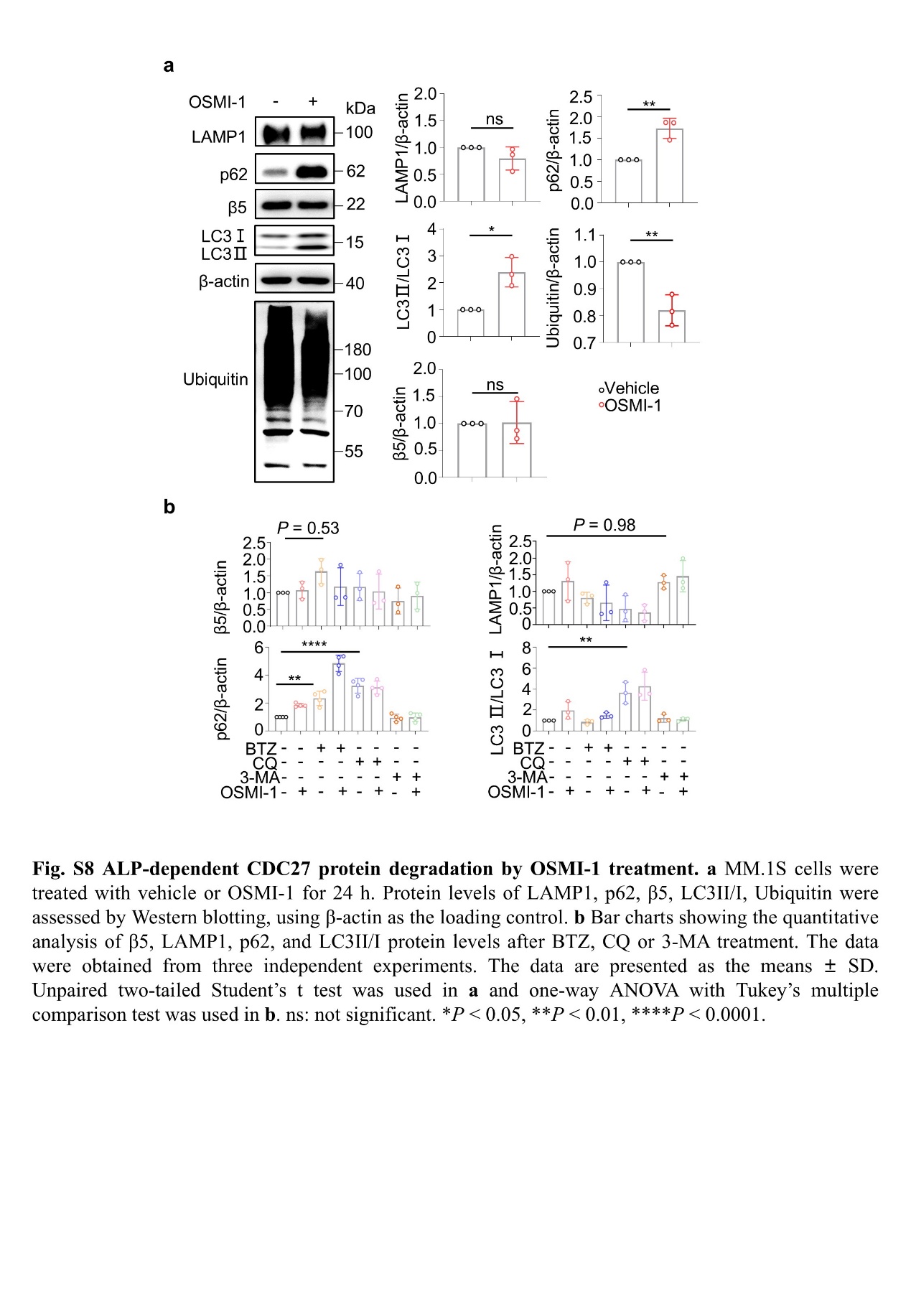


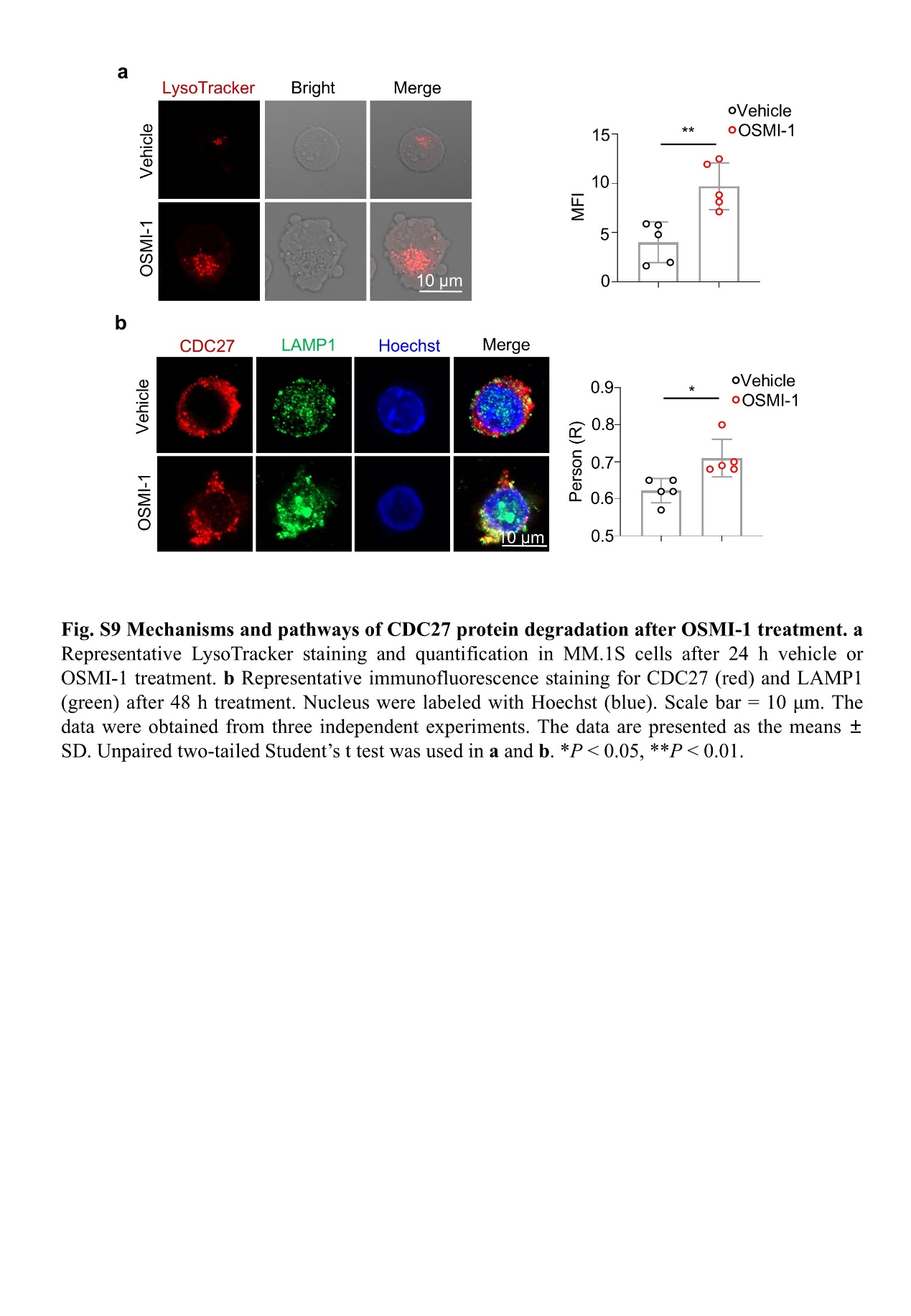


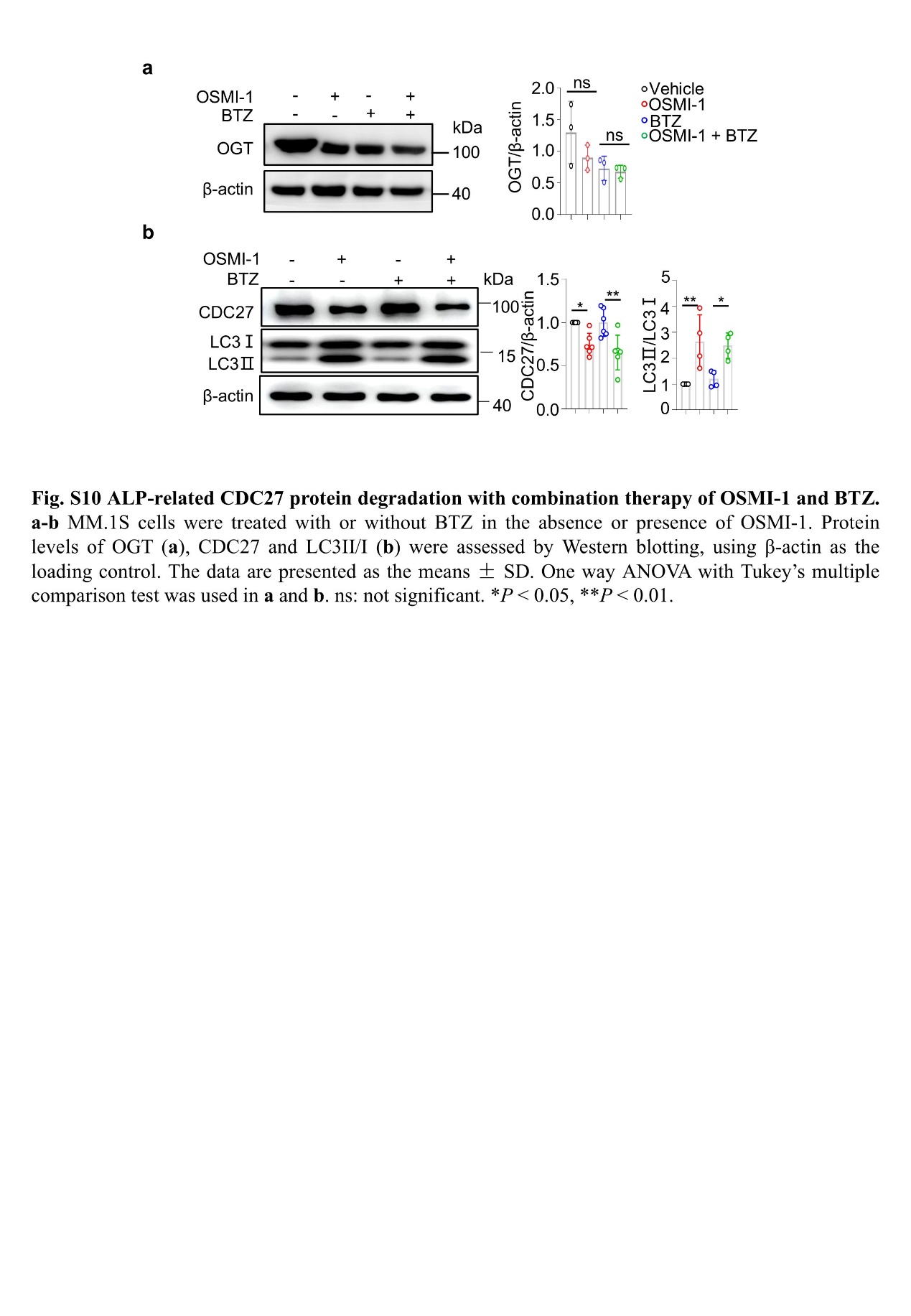


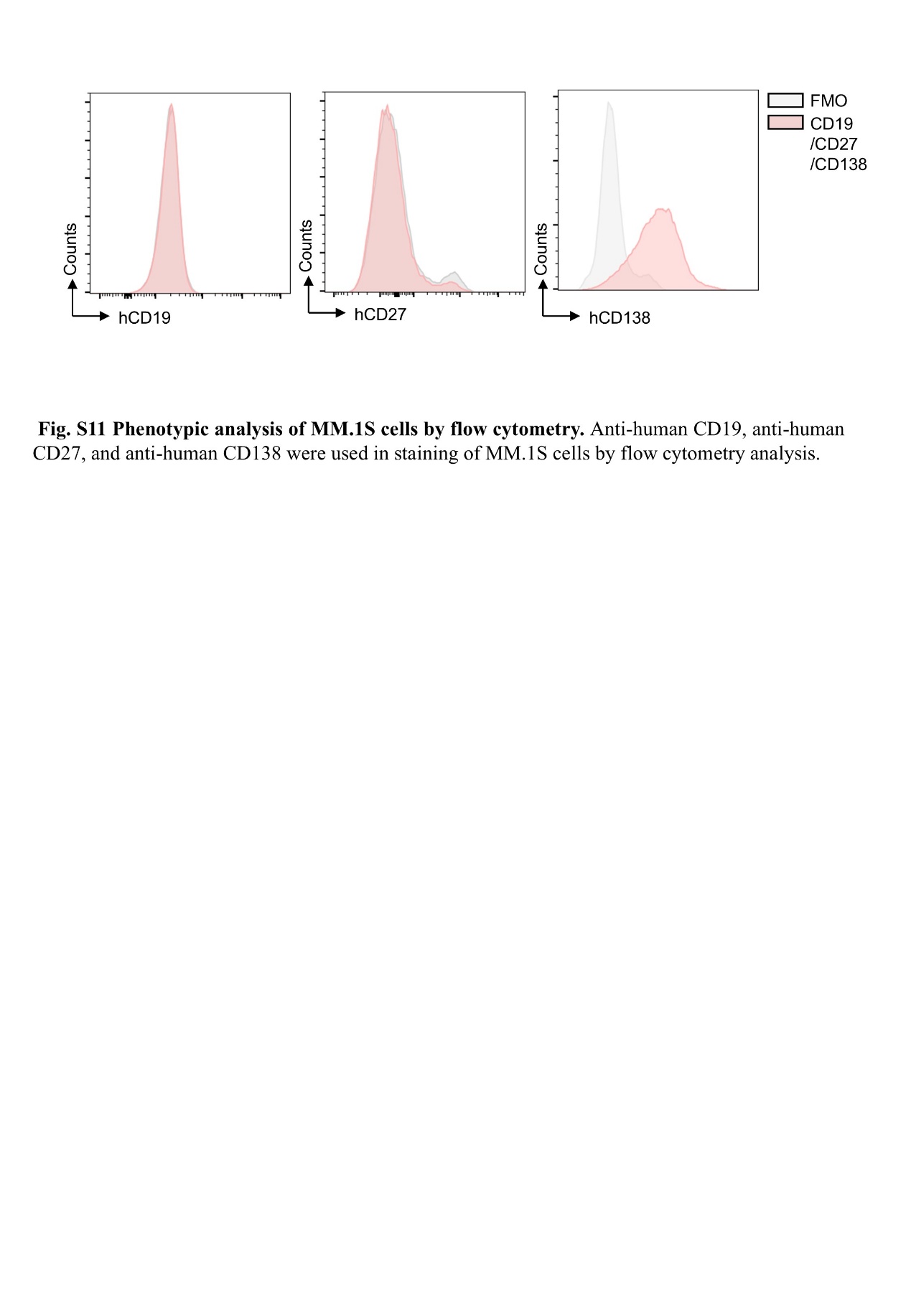


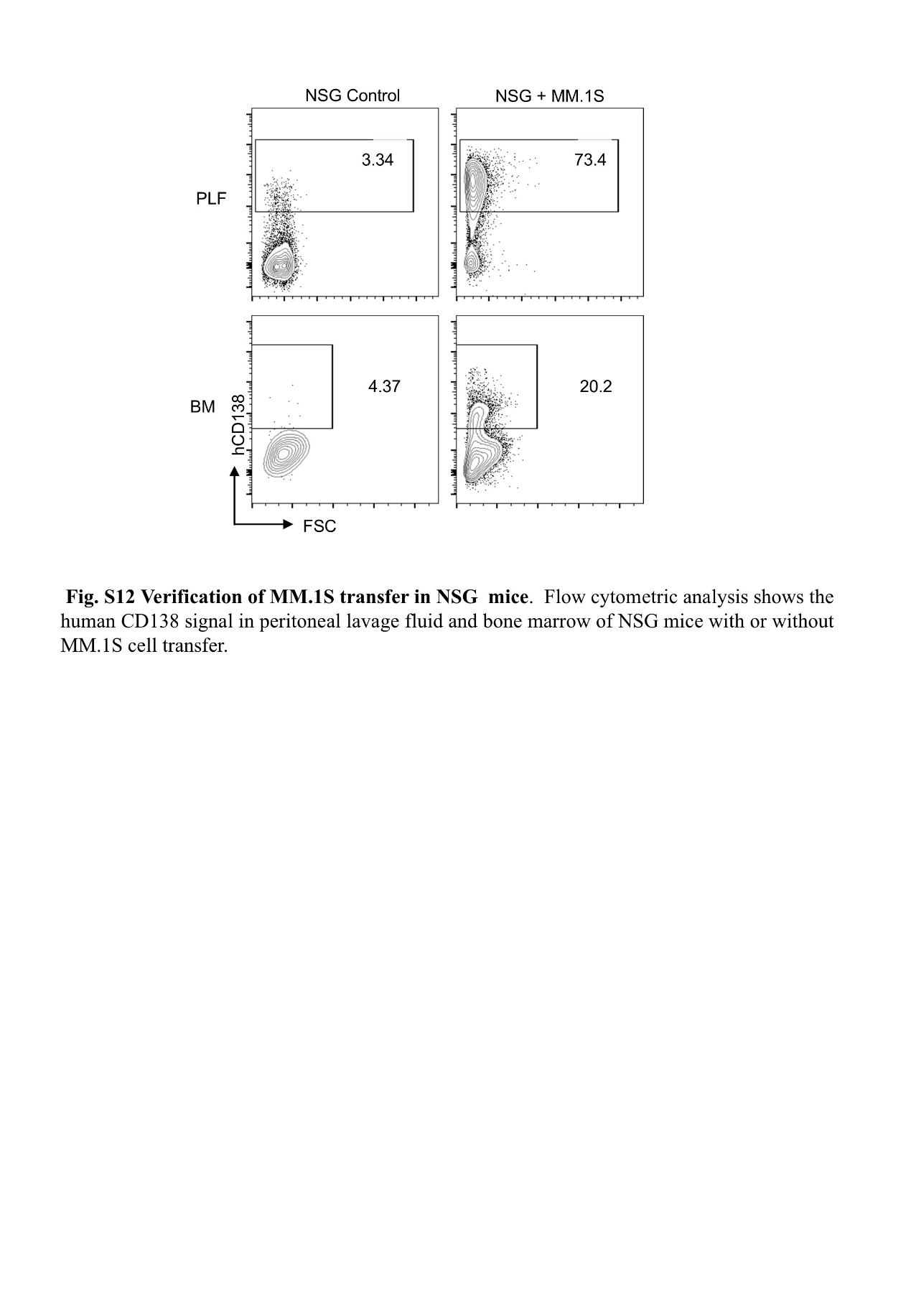

Supplement: Supplementary file 1 — Supplementary Figures S1-S12 [file 41401_2025_1500_MOESM1_ESM.docx]
