# Supplementary material for: Inhibition of CDC27 O-GlcNAcylation coordinates the antitumor efficacy in multiple myeloma through the autophagy-lysosome pathway
Source: Acta Pharmacol Sin. 2025 Feb 21;46(7):2041–55. doi: 10.1038/s41401-025-01500-2 (PMC12205061; doi:10.1038/s41401-025-01500-2)
Supplement: Supplementary file 2 — Supplementary Tables [file 41401_2025_1500_MOESM2_ESM.docx]

| Table S1. Databases used in this study | | | | | | | | |
| --- | --- | --- | --- | --- | --- | --- | --- | --- |
| Dataset | GSE5900 | GSE19784 | GSE136337 | GSE47552 | GSE39754 | GSE136324 | GSE4204 | GSE24080 |
| Sample type | Bone marrow plasma cells | Bone marrow plasma cells | Bone marrow plasma cells | Bone marrow plasma cells | Bone marrow plasma cells | Whole bone marrow | Bone marrow plasma cells | Bone marrow plasma cells |
| Experiment Type | Array | Array | Array | Array | Array | Array | Array | Array |
| Sample Size | HC: 22 | MM: 320 | MM: 426 | MM: 41 | MM: 170 | MM:867 | MM:538 | MM:559 |
|  |  |  |  | HC: 5 | HC: 6 |  |  |  |

| Table S2. Sequences for siRNA and RT-PCR in this study | | |
| --- | --- | --- |
| siRNA | | |
|  | sense | anti-sense |
| siOGT #1 | CAGUGUUGCUGAAGCAGAATT | UUCUGCUUCAGCAACACUGTT. |
| siOGT #2 | GCAUGUUAUUUGAAAGCAATT | UUGCUUUCAAAUAACAUGCTT |
| siOGT #3 | GGACAGAUUCAAAUAACAATT | UUGUUAUUUGAAUCUGUCCTT |
| siCDC27 #1 | CAGUUGUACUACACCGCAATT | UUGCGGUGUAGUACA ACUGTT |
| siCDC27 #2 | GCUGUAAUUUCACCUGAUATT | UAUCAGGUGAAAUUACAGCTT |
| siCDC27 #3 | GGUCCACAAACAAGUACAATT | UUGUACUUGUUUGUGGACCTT |
| Scramble siRNA sequence (Ctrl): | UUCUCCGAACGUGUCACGUTT | ACGUGACACGUUCGGAGAATT |
| RT-PCR | | |
| CDC27 | AGCCATCTACCTTCTCACCACTAC | GCCTTCAACTCTATAATTCTCAATCCTTC |
| β-actin | CTACAATGAGCTGCGTGTGGC | CAGGTCCAGACGCAGGATGGC |

| Table S3. Differentially expressed genes in this study | | |
| --- | --- | --- |
| 962 differentially expressed genes | | |
| Symbol | Log2FoldChange | adj.P.Val |
| KIT | 1.646094978 | 0.000475577 |
| BTBD3 | 1.630740844 | 0.000690452 |
| NDNF | 1.536680426 | 0.000381597 |
| LAMP5 | 1.451407729 | 0.015089526 |
| WNT5A | 1.284188871 | 0.002915695 |
| LAMP3 | 1.275998548 | 0.000115666 |
| H1-2 | 1.247734174 | 0.000367606 |
| CCND1 | 1.237972202 | 0.016174025 |
| PDZRN4 | 1.208025631 | 0.001257429 |
| GADD45A | 1.186927278 | 0.000167446 |
| CD200 | 1.167444767 | 0.004434905 |
| RASGRP1 | 1.140175071 | 0.015697565 |
| DDIT4 | 1.137211568 | 0.000775353 |
| PELI1 | 1.130711215 | 0.000103705 |
| RND3 | 1.12163106 | 0.025024789 |
| H2AC6 | 1.117030681 | 0.000499224 |
| CD1D | 1.11652061 | 0.002698834 |
| IER2 | 1.107754965 | 0.000216826 |
| HGF | 1.091825914 | 0.005683613 |
| DKK1 | 1.085138064 | 0.033488587 |
| GRAMD1C | 1.080024427 | 0.000931597 |
| SMAD1 | 1.072728397 | 0.028282175 |
| OGT | 1.055208564 | 2.10E-05 |
| FRZB | 1.053128046 | 0.024229865 |
| FCGR2B | 1.048362407 | 0.001026246 |
| MLLT3 | 1.04009859 | 0.000271179 |
| NLGN4X | 1.036642062 | 0.009130649 |
| HERC5 | 1.014867407 | 0.000167446 |
| TJP1 | 0.981853953 | 0.003237771 |
| CD69 | 0.980806978 | 0.047968706 |
| ESRRG | 0.977608834 | 0.002568495 |
| BAZ2B | 0.972164009 | 0.000199522 |
| TNFSF10 | 0.972083424 | 0.008249145 |
| PTP4A3 | 0.968235359 | 0.007743366 |
| CDKN1A | 0.967495435 | 2.16E-05 |
| SLTM | 0.943882087 | 0.000181046 |
| TCF4 | 0.935202371 | 0.003201681 |
| H2BC12 | 0.929858892 | 0.000181046 |
| EVI2A | 0.918614806 | 0.000199522 |
| HLTF | 0.911716611 | 0.000372161 |
| IFITM1 | 0.910858173 | 0.003127457 |
| TMEM45A | 0.898800355 | 0.018785448 |
| CHMP5 | 0.898103211 | 0.000174616 |
| UGT8 | 0.896164523 | 0.002327467 |
| MAP3K5 | 0.895052656 | 0.000319612 |
| HNRNPH2 | 0.891101289 | 0.000243268 |
| NBEA | 0.888896025 | 0.002237737 |
| NFIL3 | 0.887292856 | 0.000474188 |
| NDRG1 | 0.882235652 | 0.000167446 |
| RRAGD | 0.875936283 | 0.001014726 |
| MCC | 0.868122939 | 0.000167446 |
| ST3GAL6 | 0.866679393 | 0.000710093 |
| ST8SIA4 | 0.865309993 | 0.000299012 |
| ZFAND1 | 0.863509758 | 0.000164626 |
| CLK1 | 0.860256068 | 0.000281492 |
| MAP4K3 | 0.859187183 | 0.00158845 |
| MANSC1 | 0.854635283 | 0.010786002 |
| TOMM20 | 0.850279436 | 0.00011223 |
| VPS51 | 0.841356514 | 0.00020158 |
| USP32P2 | 0.838862932 | 0.00027269 |
| TOB1 | 0.83880074 | 0.001935949 |
| SERPINI1 | 0.835024687 | 0.017923563 |
| ZBTB20 | 0.834197317 | 0.000126129 |
| AGL | 0.830399424 | 0.001225796 |
| LSR | 0.828691113 | 0.000110615 |
| RAPGEF4 | 0.824077635 | 0.012732436 |
| RSL24D1 | 0.808438223 | 0.000288336 |
| SLC35A5 | 0.805189967 | 0.000211195 |
| CHD1 | 0.805121633 | 0.000494745 |
| SECISBP2L | 0.801784166 | 0.000145957 |
| GARS1 | 0.798111875 | 6.20E-05 |
| RPS27L | 0.793988679 | 0.000108044 |
| DBF4 | 0.792890348 | 0.000479528 |
| LAPTM4B | 0.792513389 | 0.03682288 |
| MYC | 0.791358035 | 0.025463097 |
| RNF11 | 0.787704485 | 0.000372415 |
| TMEM123 | 0.787076783 | 0.005368098 |
| ARID5A | 0.787005899 | 0.000170532 |
| IPO7 | 0.786695078 | 0.000228351 |
| CKS2 | 0.786500224 | 0.005895279 |
| RASA1 | 0.785104136 | 0.000480198 |
| HMG20A | 0.783317704 | 0.000126129 |
| SLC35A1 | 0.782821431 | 0.000245158 |
| PMEPA1 | 0.782675875 | 0.000428518 |
| BCL11A | 0.782455628 | 0.001195458 |
| RPL36 | 0.781616977 | 0.000167446 |
| TOPORS | 0.780573279 | 0.001368222 |
| ITGB7 | 0.778618797 | 0.018113236 |
| VBP1 | 0.776579131 | 0.000338965 |
| H2BC5 | 0.776212846 | 0.001935949 |
| CLIC2 | 0.77418677 | 0.028367315 |
| FBL | 0.773080249 | 0.000171435 |
| PSMB9 | 0.772884955 | 0.000167807 |
| CTR9 | 0.772479121 | 0.000541902 |
| IARS1 | 0.772271024 | 0.000229047 |
| PSAT1 | 0.771315461 | 0.003197309 |
| COX7A2 | 0.767355524 | 0.000166274 |
| CCNG1 | 0.76640687 | 0.000729976 |
| PFDN5 | 0.765144887 | 0.0001995 |
| SCYL2 | 0.76467724 | 0.007128197 |
| NECTIN3 | 0.764582875 | 0.008694512 |
| PSMB8 | 0.763846226 | 0.000115666 |
| NARS1 | 0.76231025 | 0.000228534 |
| GYG1 | 0.761884399 | 0.000158243 |
| PSMA4 | 0.760773514 | 0.000378427 |
| PRDM5 | 0.759504243 | 0.007708708 |
| FEM1C | 0.758979078 | 0.000158892 |
| KAT2B | 0.758623608 | 0.000512379 |
| RPSA | 0.757010374 | 0.000166274 |
| ARHGEF3 | 0.754521032 | 0.002667281 |
| COX6B1 | 0.754133109 | 0.000126129 |
| CCT3 | 0.753731935 | 0.000251815 |
| ACTL6A | 0.753628053 | 0.00107219 |
| CPQ | 0.752912822 | 0.000951517 |
| SERTAD2 | 0.750995771 | 0.000377387 |
| SMAD7 | 0.749722504 | 0.003036917 |
| H1-10 | 0.747867619 | 0.000269692 |
| TAF7 | 0.746410312 | 0.000120468 |
| BANF1 | 0.746371316 | 0.001042561 |
| AHCY | 0.746182901 | 0.000829451 |
| PTPRK | 0.746057921 | 0.001014726 |
| EID1 | 0.745308787 | 0.000167446 |
| RCN1 | 0.745142418 | 0.000884829 |
| NR3C1 | 0.744158981 | 0.000167446 |
| IARS2 | 0.742890611 | 0.000167446 |
| ETFA | 0.741833039 | 0.000506528 |
| FBP1 | 0.740860444 | 0.044206688 |
| LACTB2 | 0.740319719 | 0.002193159 |
| HACD2 | 0.739901362 | 0.000103705 |
| GSTP1 | 0.739598648 | 0.004022544 |
| EDF1 | 0.739277287 | 0.000125116 |
| IRAG2 | 0.739038791 | 0.006276761 |
| IFI6 | 0.736678483 | 0.040151274 |
| EVI2B | 0.735694351 | 0.000243268 |
| SEPTIN10 | 0.734176473 | 0.037933227 |
| CENPC | 0.733239273 | 0.000823778 |
| CAMLG | 0.732886136 | 0.000302544 |
| SLC1A5 | 0.732373261 | 0.00037107 |
| MTDH | 0.732138292 | 0.000411868 |
| TMEM243 | 0.731439017 | 0.000564424 |
| TSHR | 0.730801241 | 0.007032552 |
| TRIM28 | 0.730685661 | 0.000371248 |
| MRPL34 | 0.730578682 | 0.000252953 |
| SLC38A2 | 0.729587935 | 0.000337613 |
| DUSP6 | 0.729358328 | 0.026235433 |
| CTSO | 0.726834905 | 0.00046545 |
| WARS1 | 0.726074542 | 0.003777196 |
| IMPDH2 | 0.723865969 | 0.000670417 |
| TIPARP | 0.721298049 | 0.000444512 |
| NDUFB5 | 0.719291055 | 0.000347207 |
| HIKESHI | 0.719214958 | 0.000174775 |
| TOP2B | 0.717532714 | 0.001732605 |
| YIPF3 | 0.716978782 | 0.000115344 |
| PTGS2 | 0.716002967 | 0.047519656 |
| SLC7A5 | 0.715235694 | 0.004056458 |
| MRPS17 | 0.714068256 | 0.000370363 |
| HBD | 0.713951069 | 0.033488587 |
| NR1D2 | 0.713326993 | 0.00113354 |
| NME1 | 0.713207312 | 0.000332433 |
| SLIRP | 0.713055818 | 0.000389376 |
| NIT2 | 0.712316868 | 0.000255199 |
| BIRC2 | 0.712224603 | 0.001883394 |
| C1QBP | 0.711529215 | 0.000867393 |
| RCN2 | 0.71079807 | 0.000428263 |
| ITGAV | 0.710717117 | 0.001699591 |
| RABGGTB | 0.708716348 | 0.000480198 |
| TCEAL4 | 0.70832699 | 0.000216826 |
| DDT | 0.707567277 | 0.000322112 |
| TMEM35B | 0.707397632 | 0.000243268 |
| TBCA | 0.707022927 | 0.000158243 |
| MDFIC | 0.706333823 | 0.000181046 |
| RAB27A | 0.705933212 | 0.000391892 |
| LTA4H | 0.70484295 | 0.000368228 |
| GAS6 | 0.703830341 | 0.002848164 |
| NDUFA13 | 0.70369269 | 0.000313713 |
| BCAP31 | 0.703524873 | 0.000357025 |
| ICAM3 | 0.702461489 | 0.000620939 |
| GPI | 0.701474526 | 0.000677422 |
| CEBPZ | 0.701203619 | 0.000281492 |
| REEP5 | 0.700310397 | 0.000115344 |
| FAIM | 0.700041929 | 0.001332161 |
| EIF3G | 0.699739392 | 0.000164626 |
| PPA1 | 0.699639526 | 0.000370363 |
| CCNH | 0.697950549 | 0.000114282 |
| RPL35 | 0.697899816 | 0.000103705 |
| C1orf109 | 0.697862424 | 0.000520961 |
| CD180 | 0.697652538 | 0.010159015 |
| FKBP2 | 0.696940616 | 0.00015762 |
| RPL11 | 0.696750626 | 0.000170532 |
| RTRAF | 0.696470726 | 0.000167446 |
| ALCAM | 0.695552683 | 0.001044947 |
| UBL5 | 0.695363211 | 0.000167807 |
| EXOSC5 | 0.695054077 | 0.000103705 |
| TMEM38B | 0.694083597 | 0.000228065 |
| TOMM7 | 0.692725703 | 0.000115666 |
| SRP9 | 0.690628957 | 0.000411868 |
| NOL8 | 0.690465647 | 0.001337363 |
| MCUB | 0.69038723 | 0.001116985 |
| DMTF1 | 0.69017675 | 0.000203205 |
| TRMT11 | 0.689763286 | 0.001494762 |
| ANP32B | 0.689711496 | 0.000115666 |
| TMEM14B | 0.68850934 | 0.000348129 |
| RNF111 | 0.687678365 | 0.001853189 |
| SECISBP2 | 0.684319859 | 0.000184595 |
| PKD2 | 0.682822525 | 0.001518078 |
| PSMB3 | 0.682775937 | 0.000199522 |
| SET | 0.681973169 | 0.00015232 |
| RHOH | 0.681876211 | 0.002922854 |
| CNTN1 | 0.681439442 | 0.027536961 |
| MAN2A1 | 0.681426783 | 0.001056662 |
| TMA16 | 0.680608744 | 0.011855162 |
| NDUFA4 | 0.680352862 | 0.000238968 |
| NAP1L1 | 0.679652929 | 0.000262672 |
| H2BC6 | 0.679134037 | 0.003185989 |
| ALG8 | 0.67911031 | 0.000266764 |
| BST2 | 0.678920484 | 0.000511238 |
| PPP1CC | 0.67837255 | 0.000351076 |
| HSD17B11 | 0.678033316 | 0.004515243 |
| DYNLL1 | 0.677060776 | 0.001016298 |
| ATP5MG | 0.67615731 | 0.000198085 |
| PCYOX1 | 0.67592237 | 0.002522181 |
| H2BC21 | 0.67568871 | 0.018209891 |
| MYO5C | 0.674516556 | 0.003960657 |
| EPRS1 | 0.673063646 | 0.000698368 |
| GGH | 0.672579538 | 0.023482189 |
| PFN2 | 0.671367399 | 0.010826186 |
| PHGDH | 0.671197009 | 0.000573172 |
| RPL4 | 0.671121287 | 0.000103705 |
| UBE2E3 | 0.670784343 | 0.000153842 |
| PRMT3 | 0.670505476 | 0.003251665 |
| CCPG1 | 0.670286801 | 0.001509899 |
| DUSP5 | 0.668778031 | 0.012399492 |
| LSM7 | 0.668670697 | 0.001879878 |
| TRMT112 | 0.668395055 | 0.000159926 |
| LARS1 | 0.668376309 | 0.000136809 |
| LAMC1 | 0.668275082 | 0.000899839 |
| SQOR | 0.667499889 | 0.000167446 |
| MSMO1 | 0.666169989 | 0.006115904 |
| JUNB | 0.66586884 | 0.002786112 |
| SIDT1 | 0.665682196 | 0.004246344 |
| DERA | 0.665362776 | 0.001989777 |
| SH3BP5 | 0.665153411 | 0.008037032 |
| C2CD5 | 0.665035345 | 0.001388672 |
| PSMB4 | 0.664974763 | 0.000153371 |
| SARAF | 0.664071822 | 0.000863769 |
| SEC22B | 0.66327348 | 0.000255199 |
| ECI1 | 0.662862013 | 0.000126129 |
| H2BC7 | 0.662739866 | 0.002358009 |
| PFKM | 0.662449472 | 0.000550557 |
| OXR1 | 0.661687723 | 0.000243268 |
| CLIC1 | 0.661574483 | 0.000158243 |
| CAAP1 | 0.661053367 | 0.000467083 |
| HSP90AA1 | 0.660311635 | 0.000612639 |
| TNFRSF17 | 0.656798114 | 0.002530125 |
| SATB1 | 0.656721628 | 0.00989462 |
| C21orf91 | 0.656444771 | 0.002131903 |
| RPS25 | 0.656324991 | 0.000141202 |
| CBX7 | 0.655805306 | 0.000398621 |
| TVP23B | 0.655713129 | 0.002610455 |
| CXCR4 | 0.655184348 | 0.014466667 |
| SNRPD2 | 0.654995477 | 0.000370363 |
| ARPC5L | 0.654702647 | 0.000345435 |
| RALGPS2 | 0.653979638 | 0.00160442 |
| TNFRSF13B | 0.653886563 | 0.000281492 |
| HLA-F | 0.653087078 | 0.00016091 |
| RBMS1 | 0.652864519 | 0.000709154 |
| HS2ST1 | 0.652361529 | 0.003956926 |
| LRIF1 | 0.651251351 | 0.008154334 |
| IRF9 | 0.650661698 | 0.000665801 |
| RAP2C | 0.650133574 | 0.002073378 |
| CNIH1 | 0.649724996 | 0.000457243 |
| PRKCB | 0.649550514 | 0.00298176 |
| DNAJC1 | 0.649351135 | 0.000259934 |
| NDUFAB1 | 0.648625766 | 0.000115666 |
| HSPA1A | 0.648081534 | 0.042648417 |
| MRPL22 | 0.647940973 | 0.000579426 |
| LDHA | 0.647293631 | 0.000243268 |
| PRKD3 | 0.646798708 | 0.003546568 |
| SON | 0.64622095 | 0.000181046 |
| IL6R | 0.645797617 | 0.011905862 |
| ADGRE5 | 0.645540474 | 0.001813681 |
| ANKRA2 | 0.645431572 | 0.000944248 |
| DNAJA1 | 0.645014885 | 0.000302544 |
| BTAF1 | 0.644417207 | 0.007732604 |
| SERINC1 | 0.642862544 | 0.002537077 |
| HMGN3 | 0.640795237 | 0.000602262 |
| CERK | 0.640051129 | 0.000379932 |
| QARS1 | 0.639656048 | 0.000377387 |
| AVEN | 0.639467183 | 0.000356537 |
| DENND4A | 0.63873939 | 0.000913329 |
| RAN | 0.638725461 | 0.000404115 |
| MACIR | 0.638655863 | 0.002091837 |
| UTP3 | 0.638597078 | 0.001350552 |
| NTAN1 | 0.638273767 | 0.003487372 |
| GPX7 | 0.638246779 | 0.001511823 |
| SRI | 0.635971663 | 0.000464174 |
| EMC2 | 0.635023087 | 0.005000564 |
| RPL36AL | 0.634597174 | 0.000554742 |
| PSMB6 | 0.633530578 | 0.000221883 |
| NDUFAF1 | 0.633263412 | 0.003049139 |
| ERAP2 | 0.6331547 | 0.043014095 |
| THOC7 | 0.632051385 | 0.002493523 |
| HNRNPAB | 0.632021607 | 0.0001995 |
| STT3A | 0.631853281 | 0.002413597 |
| INTS8 | 0.631261701 | 0.000411868 |
| SSR2 | 0.631067204 | 0.000223944 |
| CUTA | 0.630500983 | 0.000132435 |
| GPX4 | 0.630406737 | 0.000402932 |
| SLCO5A1 | 0.630124025 | 0.008159427 |
| STOML2 | 0.629913072 | 0.000520961 |
| CNBP | 0.629519327 | 0.000103705 |
| LAPTM4A | 0.629270462 | 0.000444512 |
| TPRKB | 0.629157819 | 0.001905595 |
| NTAQ1 | 0.62905104 | 0.000659357 |
| EIF3L | 0.628560779 | 0.00029102 |
| ARMCX3 | 0.628047545 | 0.002922854 |
| BBS10 | 0.627830009 | 0.003231472 |
| RRS1 | 0.627380679 | 0.000370363 |
| VPS37B | 0.627065974 | 0.000909709 |
| HCP5 | 0.626596918 | 0.005866599 |
| NDUFS5 | 0.626503899 | 0.000383061 |
| TALDO1 | 0.626449496 | 0.000398621 |
| IMP3 | 0.626360187 | 0.000243268 |
| NDUFAF4 | 0.626215617 | 0.000913329 |
| NDUFS6 | 0.626073666 | 0.000176302 |
| TMEM147 | 0.625974565 | 0.000590469 |
| SLAMF1 | 0.625306457 | 0.040435524 |
| CD55 | 0.625180737 | 0.001629872 |
| LSM5 | 0.624930757 | 0.003142486 |
| NDUFA3 | 0.624810324 | 0.000158316 |
| GLB1 | 0.624555974 | 0.001420742 |
| KCTD3 | 0.624508334 | 0.008128004 |
| CYP51A1 | 0.624321908 | 0.00030375 |
| CRY1 | 0.624226476 | 0.018293801 |
| BTG1 | 0.624063536 | 0.005356495 |
| BTK | 0.624034658 | 0.000448976 |
| BAZ1A | 0.623848972 | 0.005163331 |
| ZFP36 | 0.623590768 | 0.000399565 |
| EIF3D | 0.62343742 | 0.000447105 |
| PARM1 | 0.622612168 | 0.01851078 |
| FKBP5 | 0.622487614 | 0.014795539 |
| IFI16 | 0.622105409 | 0.008374728 |
| PSMD2 | 0.622077885 | 0.000108044 |
| FAU | 0.621710013 | 0.000103705 |
| SUCLG2 | 0.62149479 | 0.002561198 |
| DYNLT1 | 0.621450173 | 0.002017196 |
| CCT7 | 0.620882294 | 0.000173844 |
| ATP5F1D | 0.620042346 | 0.000243268 |
| CEBPB | 0.61987395 | 0.000863445 |
| TAX1BP1 | 0.619840721 | 0.000522774 |
| RPL22 | 0.619431839 | 0.000164626 |
| PPIP5K2 | 0.619155174 | 0.004186077 |
| RETREG3 | 0.618965466 | 0.000284155 |
| SPINT2 | 0.618518688 | 0.044325244 |
| CDC27 | 0.616889073 | 1.24E-05 |
| CEBPG | 0.616755193 | 0.000354878 |
| CCDC69 | 0.616481657 | 0.00188047 |
| H2AZ1 | 0.616312172 | 0.000841854 |
| EPS15 | 0.615959569 | 0.000995165 |
| PAIP1 | 0.615796989 | 0.000208362 |
| TRIAP1 | 0.615351998 | 0.000103705 |
| AUH | 0.615326714 | 0.001225016 |
| UQCRFS1 | 0.615192415 | 0.000322574 |
| TNFSF8 | 0.614846209 | 0.017157579 |
| SLC25A20 | 0.614764105 | 0.000991955 |
| LDHB | 0.614476827 | 0.000769275 |
| ADRB2 | 0.614321401 | 0.006599244 |
| RPS21 | 0.614186165 | 0.000103705 |
| EEF2 | 0.613906155 | 0.0002036 |
| UAP1 | 0.613696487 | 0.001986134 |
| KLHL24 | 0.613653026 | 0.000411868 |
| COX8A | 0.613570799 | 0.000103705 |
| ASNSD1 | 0.612853347 | 0.000442287 |
| HEXB | 0.612686866 | 0.00225973 |
| TERF1 | 0.612523171 | 0.000670417 |
| ATF6 | 0.612274497 | 0.001183498 |
| ADNP | 0.612148545 | 0.000863769 |
| COX6C | 0.612047089 | 0.000413731 |
| CAV2 | 0.61188034 | 0.002786112 |
| SAMSN1 | 0.611660764 | 0.020291196 |
| COX7B | 0.611631649 | 0.000667389 |
| H2AC11 | 0.611566026 | 0.015945373 |
| EIF3J | 0.611368231 | 0.000520961 |
| UBAP2 | 0.611351221 | 0.00016091 |
| THG1L | 0.611257587 | 0.003956926 |
| CTNNA2 | 0.611167599 | 0.019398812 |
| LPIN1 | 0.610653618 | 0.000452941 |
| SARS1 | 0.610463557 | 0.000393911 |
| SMAD5 | 0.610266028 | 0.00022069 |
| EIF1AX | 0.609994671 | 0.000861399 |
| CD46 | 0.60953219 | 0.002796782 |
| IFITM2 | 0.609331169 | 0.0039975 |
| SNHG32 | 0.608122585 | 0.001370307 |
| LONP1 | 0.6078779 | 0.000115666 |
| TBCCD1 | 0.607671679 | 0.000391892 |
| ZNF146 | 0.6072892 | 0.000367157 |
| CTNNAL1 | 0.60707891 | 0.000469663 |
| GANAB | 0.606949773 | 0.000243268 |
| TSPYL4 | 0.606852726 | 0.003414835 |
| ALAS1 | 0.606842438 | 0.000208362 |
| ATIC | 0.60550993 | 0.000355931 |
| TM2D3 | 0.605268297 | 0.000848499 |
| MRPL13 | 0.60508137 | 0.003163021 |
| SELENOT | 0.604191694 | 0.000901983 |
| RBX1 | 0.604131355 | 0.000825088 |
| NDUFA1 | 0.603468925 | 0.000243268 |
| RECQL | 0.603337426 | 0.003548768 |
| TMEM14A | 0.603279018 | 0.004903835 |
| ACVR1 | 0.603110119 | 0.002788953 |
| STAT4 | 0.602852791 | 0.006105284 |
| DECR1 | 0.60196196 | 0.000697768 |
| KARS1 | 0.601049106 | 0.000219728 |
| GBE1 | 0.600963819 | 0.002184692 |
| STK17A | 0.599942428 | 0.003898162 |
| DPAGT1 | 0.599647985 | 0.000258769 |
| ATP5PF | 0.599507 | 0.000313713 |
| CYSLTR1 | 0.59928181 | 0.000926676 |
| CETN3 | 0.59920871 | 0.012047293 |
| RPL15 | 0.59876818 | 0.000141277 |
| NDUFA8 | 0.598749837 | 0.001170508 |
| MRPL24 | 0.598499625 | 0.000543964 |
| BBC3 | 0.598329082 | 0.000103705 |
| ZNF721 | 0.597567916 | 0.0021312 |
| MOAP1 | 0.597531637 | 0.000873622 |
| CADPS2 | 0.597070561 | 0.020638898 |
| USP1 | 0.596488783 | 0.003474422 |
| TMEM168 | 0.595758188 | 0.00029476 |
| CYC1 | 0.595170424 | 0.000400267 |
| TAF1D | 0.595149386 | 0.003544701 |
| ELK3 | 0.595080409 | 0.000266285 |
| LIN7C | 0.595007297 | 0.001149651 |
| SKIC3 | 0.59425969 | 0.003590395 |
| P2RX5 | 0.594104942 | 0.019267166 |
| ARMT1 | 0.594001049 | 0.018661555 |
| TRAPPC8 | 0.593729395 | 0.001000606 |
| TMX1 | 0.593472504 | 0.002148467 |
| SOD1 | 0.5934519 | 0.000646593 |
| PIGK | 0.593052563 | 0.000506528 |
| SKP1 | 0.592808834 | 0.000185517 |
| SNRPA1 | 0.592683286 | 0.000480198 |
| NDUFS8 | 0.592558509 | 0.000103705 |
| TSPAN31 | 0.592431033 | 0.000325891 |
| AEN | 0.591815249 | 0.000137417 |
| SSR3 | 0.591256295 | 0.001034647 |
| PIGF | 0.591114166 | 0.000986323 |
| YIF1A | 0.591037348 | 0.000779326 |
| PRKCSH | 0.59091127 | 0.000853367 |
| PILRB | 0.589563764 | 0.000372161 |
| SENP6 | 0.58943999 | 0.001021625 |
| NEK7 | 0.589195107 | 0.012289323 |
| PIM2 | 0.588578542 | 0.005298632 |
| UBA52 | 0.588577433 | 0.000103705 |
| MINDY2 | 0.588519175 | 0.000694178 |
| SMARCA5 | 0.588170598 | 0.000759096 |
| HSPA8 | 0.588154655 | 0.000398621 |
| BTG2 | 0.588054201 | 0.001328019 |
| THUMPD1 | 0.588002569 | 0.001366895 |
| LETMD1 | 0.587909692 | 0.000590831 |
| RPA3 | 0.587232128 | 0.000604398 |
| ATP5F1B | 0.586966461 | 0.00018479 |
| PRPF8 | 0.586760644 | 0.00030375 |
| PGRMC2 | 0.586573968 | 0.001000606 |
| ITM2A | 0.586519702 | 0.040864403 |
| SOCS5 | 0.58640324 | 0.000362404 |
| RSL1D1 | 0.586399965 | 0.001144943 |
| SND1 | 0.586107974 | 0.000103705 |
| CSK | 0.585840478 | 0.00030375 |
| S1PR4 | 0.585396939 | 0.001769363 |
| NR4A2 | 0.585120889 | 0.034001616 |
| SMC4 | 0.584943905 | 0.002807706 |
| MRPS28 | 0.584698365 | 0.003430719 |
| SINHCAF | 0.584210035 | 0.011941075 |
| NFU1 | 0.584102776 | 0.001146758 |
| TMEM248 | 0.583288097 | 0.000170532 |
| ZNF711 | 0.582662456 | 0.010359198 |
| EEF1B2 | 0.582324712 | 0.000145957 |
| SEC62 | 0.582218363 | 0.002394231 |
| NIPBL | 0.581936752 | 0.001010637 |
| EIF3A | 0.581819761 | 0.00070995 |
| PPP3CA | 0.581673309 | 0.001411637 |
| POP5 | 0.581606193 | 0.000961726 |
| CKLF | 0.580990285 | 0.005622416 |
| CTSF | 0.580968696 | 0.005325812 |
| RPL30 | 0.580942299 | 0.000103705 |
| ALG3 | 0.580913234 | 0.000452941 |
| SYPL1 | 0.580831284 | 0.048224937 |
| GAREM1 | 0.580650077 | 0.02245112 |
| RBM7 | 0.579908351 | 0.002148467 |
| DARS1 | 0.579572793 | 0.00216923 |
| KDSR | 0.579567333 | 0.000360446 |
| DPF2 | 0.579160745 | 0.000305693 |
| CDC23 | 0.578758572 | 0.003710438 |
| ZNF106 | 0.578508873 | 0.000165677 |
| IGFLR1 | 0.57803381 | 0.002151142 |
| EIF4A1 | 0.57779897 | 0.000564277 |
| SCRN1 | 0.577749052 | 0.003495337 |
| NEB | 0.577615409 | 0.037933227 |
| INSR | 0.577468912 | 0.000325891 |
| FBXL4 | 0.57719987 | 0.001420742 |
| UBE4A | 0.576970532 | 0.001175037 |
| MRPL48 | 0.576880646 | 0.000198085 |
| MTERF3 | 0.576623461 | 0.003550901 |
| TBCB | 0.576158736 | 0.000271179 |
| CPOX | 0.576086095 | 0.003917382 |
| C19orf53 | 0.576070904 | 0.000204242 |
| BBX | 0.576030703 | 0.000522774 |
| LAP3 | 0.576018108 | 0.00608437 |
| DROSHA | 0.575714241 | 0.000949518 |
| GLRX | 0.575245882 | 0.010271616 |
| RFK | 0.575063886 | 0.00091505 |
| PRC1 | 0.57505948 | 0.013437537 |
| DDHD2 | 0.574743396 | 0.002585097 |
| MAGT1 | 0.574326619 | 0.001443157 |
| CAPN2 | 0.574108868 | 0.000451755 |
| TLE1 | 0.574060458 | 0.005414639 |
| EIF4H | 0.573992691 | 0.000266764 |
| KCNN3 | 0.573973786 | 0.001291257 |
| TMEM268 | 0.57390358 | 0.000990531 |
| NIPA2 | 0.573367247 | 0.000214747 |
| RANBP2 | 0.573268124 | 0.000496714 |
| ATRAID | 0.573167556 | 0.000513421 |
| MRPL16 | 0.57293958 | 0.000367157 |
| SEC24B | 0.572709123 | 0.004604505 |
| POU2AF1 | 0.572413475 | 0.000251815 |
| GALNT3 | 0.572397414 | 0.002394231 |
| STOM | 0.572249437 | 0.001695382 |
| MTIF2 | 0.571994991 | 0.002445056 |
| TAF12 | 0.571884646 | 0.000642998 |
| LYRM1 | 0.571769002 | 0.001662605 |
| RPL23AP32 | 0.571201677 | 0.000255199 |
| ACYP2 | 0.570855834 | 0.001036998 |
| UXT | 0.570838672 | 0.000686245 |
| KPNA2 | 0.570793496 | 0.005588481 |
| DDX21 | 0.570407243 | 0.00123801 |
| NDUFB11 | 0.570296181 | 0.000355931 |
| JUND | 0.569702504 | 0.000550557 |
| ARHGAP5 | 0.569554182 | 0.00039594 |
| RRP15 | 0.569453952 | 0.0016045 |
| PNP | 0.569367558 | 0.015504855 |
| UGCG | 0.568729428 | 0.003246153 |
| LAMP2 | 0.568607368 | 0.002394231 |
| COPS2 | 0.568484477 | 0.002298285 |
| SIGMAR1 | 0.568313522 | 0.000388265 |
| HSPA9 | 0.568296321 | 8.83E-05 |
| SLC25A32 | 0.568215359 | 0.002998786 |
| PAPSS1 | 0.567308887 | 0.001715116 |
| CERT1 | 0.566917531 | 0.000715172 |
| MDK | 0.566915414 | 0.011927657 |
| CCDC6 | 0.566770172 | 0.009823541 |
| MKKS | 0.566727817 | 0.000411868 |
| ERP29 | 0.565958255 | 0.000440681 |
| EXOC1 | 0.565945811 | 0.005767136 |
| ANKRD36B | 0.565792587 | 0.04507607 |
| DPM3 | 0.565683925 | 0.009607938 |
| IGF2R | 0.565609451 | 0.000404257 |
| CCDC47 | 0.565485878 | 0.001303001 |
| GATD3 | 0.565478706 | 0.00016091 |
| EMC7 | 0.565369365 | 0.000431626 |
| GABARAPL3 | 0.565338168 | 0.000262672 |
| CCT6A | 0.565288837 | 0.002545483 |
| SLC38A1 | 0.565096519 | 0.000324528 |
| PSME2 | 0.564665182 | 0.001221949 |
| HACD3 | 0.564209955 | 0.000990531 |
| COPS6 | 0.564046273 | 0.000103705 |
| CLPP | 0.563929504 | 0.000164626 |
| CYRIA | 0.563916563 | 0.021314329 |
| TOP1 | 0.563733946 | 0.009958616 |
| NUDT9 | 0.563209703 | 0.000389376 |
| ATP1A1 | 0.563207498 | 0.000667389 |
| RAB33B | 0.563167613 | 0.001934091 |
| EPS8 | 0.563085706 | 0.049509208 |
| EIF3K | 0.562998948 | 0.000158892 |
| YPEL5 | 0.562808955 | 0.001771617 |
| EMP3 | 0.562392887 | 0.047403802 |
| IDH2 | 0.561924069 | 0.004031121 |
| POGLUT1 | 0.561812863 | 0.001838456 |
| ODC1 | 0.561758419 | 0.001188417 |
| ABHD17A | 0.561660238 | 0.000228065 |
| PHF1 | 0.561503387 | 0.000188863 |
| MFAP3L | 0.56128531 | 0.012077132 |
| JADE1 | 0.561251489 | 0.000411868 |
| WIPI1 | 0.560928379 | 0.003813832 |
| LAMTOR5 | 0.560900595 | 0.000453671 |
| CRBN | 0.560503017 | 0.000791095 |
| VPS4B | 0.560113902 | 0.005174441 |
| CYP20A1 | 0.560032659 | 0.002403958 |
| TENT4A | 0.559713604 | 0.002295243 |
| PSMG1 | 0.559485544 | 0.002922854 |
| THYN1 | 0.55941557 | 0.000524164 |
| GOLT1B | 0.558986031 | 0.00274181 |
| TRIM52 | 0.558941448 | 0.00105934 |
| UBR5 | 0.558243665 | 0.003566953 |
| IGF1 | 0.558236119 | 0.000705717 |
| EEF1D | 0.558179805 | 0.000115666 |
| REX1BD | 0.558087463 | 0.00034777 |
| ZNF593 | 0.558085433 | 0.000756391 |
| CD47 | 0.557943439 | 0.000103705 |
| RINT1 | 0.557481179 | 0.008653588 |
| TDP2 | 0.557366502 | 0.005503403 |
| ALG9 | 0.55716207 | 0.000520961 |
| NOP53 | 0.557151528 | 0.000199522 |
| DNAJB14 | 0.557055368 | 0.007838133 |
| SLC35D2 | 0.556855356 | 0.002444393 |
| SNRPF | 0.556721193 | 0.000708648 |
| USP16 | 0.556609349 | 0.001643616 |
| GMPR | 0.556505983 | 0.003116723 |
| SLC3A2 | 0.556416483 | 0.002484882 |
| BAG1 | 0.556381748 | 0.000103705 |
| ALOX5 | 0.556320852 | 0.006234898 |
| KLF2 | 0.556197336 | 0.002032112 |
| EIF5 | 0.556158038 | 0.000703172 |
| DCXR | 0.556151477 | 0.000302212 |
| CD59 | 0.555542781 | 0.001370307 |
| P2RX1 | 0.555491048 | 0.040714956 |
| EI24 | 0.55535318 | 0.00115844 |
| POLR2K | 0.555067768 | 0.001446587 |
| CEP350 | 0.554876479 | 0.00187466 |
| UBXN7 | 0.554596423 | 0.000823808 |
| POLR2J | 0.553888815 | 0.000475527 |
| CHMP2B | 0.55377311 | 0.00127601 |
| FAM162A | 0.553037933 | 0.000115666 |
| C12orf29 | 0.552879214 | 0.00057931 |
| PABPC3 | 0.55275708 | 0.000243268 |
| IL6ST | 0.552260269 | 0.001589504 |
| GNE | 0.552237959 | 0.001629872 |
| FDPS | 0.552142114 | 0.000708023 |
| AHSA1 | 0.55211079 | 0.000255199 |
| PSMD14 | 0.551757323 | 0.002051219 |
| ECI2 | 0.551680698 | 0.000873622 |
| COPB2 | 0.551675678 | 0.003654817 |
| CASP3 | 0.550897033 | 0.018153147 |
| QTRT1 | 0.550728129 | 0.000486884 |
| ADAR | 0.550270798 | 0.000612639 |
| DUT | 0.550205983 | 0.000452941 |
| PUS3 | 0.549856261 | 0.001436505 |
| ZNF227 | 0.54981869 | 0.003224855 |
| SEC61A1 | 0.549784992 | 0.001376042 |
| ANP32A | 0.549691092 | 0.000463133 |
| HSPA5 | 0.549602159 | 0.000604847 |
| METTL7A | 0.54948724 | 0.000201896 |
| CSDE1 | 0.549461067 | 0.002597533 |
| ARMCX2 | 0.54915096 | 0.022993639 |
| NLK | 0.548977316 | 0.002316458 |
| SIL1 | 0.548899262 | 0.001281627 |
| UBE2E1 | 0.548115272 | 0.001016752 |
| SNRPD1 | 0.548105165 | 0.000411868 |
| SLBP | 0.548092353 | 0.004653283 |
| ASNS | 0.548076026 | 0.000386474 |
| AREG | 0.547677819 | 0.022237288 |
| PSMA1 | 0.547660551 | 0.000854669 |
| BMPR2 | 0.547471025 | 0.000543876 |
| PMVK | 0.54735579 | 0.000493142 |
| TPR | 0.547056874 | 0.000864652 |
| XPOT | 0.54693334 | 0.002966572 |
| MRPL46 | 0.546819357 | 0.000411868 |
| FMR1 | 0.546805813 | 0.021187307 |
| SMNDC1 | 0.546657602 | 0.001589047 |
| ADAM10 | 0.546570493 | 0.004214034 |
| NFE2L1 | 0.545826936 | 0.001111445 |
| COPZ1 | 0.545702373 | 0.000348862 |
| TMBIM6 | 0.545401891 | 0.000678451 |
| HSP90AB1 | 0.545122169 | 0.006105415 |
| MFAP1 | 0.54510433 | 0.001902119 |
| LUC7L3 | 0.545050738 | 0.00097085 |
| RPL14 | 0.544494624 | 0.000158892 |
| COPS5 | 0.544466502 | 0.00265048 |
| ENOPH1 | 0.543936899 | 0.001411637 |
| RPS19 | 0.543878697 | 1.74E-05 |
| BAX | 0.543768611 | 0.000950809 |
| NOL11 | 0.54365789 | 0.00406023 |
| MSANTD2 | 0.543526053 | 0.00568979 |
| LDLRAP1 | 0.543508109 | 0.00027269 |
| GOLPH3 | 0.543007565 | 0.001809868 |
| ALDH9A1 | 0.542949298 | 0.00136488 |
| PSMB5 | 0.542872233 | 0.00046545 |
| ZNF706 | 0.54277615 | 0.001017009 |
| PARP1 | 0.542599941 | 0.000582859 |
| SELENOF | 0.542423612 | 0.004591876 |
| RRM1 | 0.542340085 | 0.00052028 |
| PSMC5 | 0.542318688 | 0.000368631 |
| UBE2L6 | 0.542287925 | 0.001791538 |
| KRIT1 | 0.54227447 | 0.000714658 |
| ZNF654 | 0.541801493 | 0.001779311 |
| DMXL1 | 0.541431636 | 0.003030983 |
| GMFG | 0.541418149 | 0.00575474 |
| SMIM14 | 0.541195128 | 0.005776874 |
| BPTF | 0.540969309 | 0.000949518 |
| UBA5 | 0.540926638 | 0.000904242 |
| NAPA | 0.540863249 | 0.002697848 |
| NOP14 | 0.540773594 | 0.00026859 |
| SUCLA2 | 0.540486999 | 0.018621529 |
| GTF2B | 0.540368902 | 0.0018984 |
| DNMT1 | 0.540328927 | 0.005826274 |
| GABARAPL1 | 0.539948797 | 0.001804198 |
| TOR3A | 0.539947779 | 0.001199357 |
| TSG101 | 0.539767684 | 0.000438793 |
| PIGH | 0.539765528 | 0.002751275 |
| PPP6C | 0.539524175 | 0.000250272 |
| MDH2 | 0.539017173 | 0.0002225 |
| UNC50 | 0.538989109 | 0.000752874 |
| SIRT1 | 0.538980715 | 0.006490625 |
| BABAM1 | 0.538928704 | 0.00043811 |
| MYO6 | 0.538694762 | 0.017287792 |
| EPM2AIP1 | 0.538670459 | 0.000386819 |
| HAUS3 | 0.538518487 | 0.008362897 |
| DBI | 0.538486042 | 0.001629872 |
| ATP6V0B | 0.538369174 | 0.001425876 |
| SSNA1 | 0.538320198 | 0.000170532 |
| UGDH | 0.53814583 | 0.003956926 |
| PSMB10 | 0.537339248 | 0.00348875 |
| TAPBPL | 0.537190016 | 0.004787329 |
| FXYD5 | 0.537140156 | 0.000861399 |
| DNASE2 | 0.53703214 | 0.000660948 |
| HIBCH | 0.536952071 | 0.01162028 |
| ATP5MJ | 0.536904472 | 0.001111445 |
| RIOK2 | 0.536571731 | 0.003728519 |
| PSMA2 | 0.536534844 | 0.001367062 |
| RPS6KA3 | 0.536410111 | 0.001623515 |
| UBR7 | 0.536213641 | 0.00187466 |
| SEC63 | 0.535974033 | 0.00157837 |
| RPS2 | 0.535903566 | 0.000874145 |
| NCBP2 | 0.535840505 | 0.000302544 |
| HINT1 | 0.535436071 | 0.000861399 |
| AP2M1 | 0.535120334 | 0.002394231 |
| CSRP1 | 0.535020977 | 0.000199522 |
| ANXA7 | 0.534774568 | 0.002163777 |
| PLOD3 | 0.534284229 | 0.000398621 |
| TMCO1 | 0.534225558 | 0.001977283 |
| MBD4 | 0.534076378 | 0.001974403 |
| WDR82 | 0.533803615 | 0.000371248 |
| TSNAX | 0.533652038 | 0.008029468 |
| PSME1 | 0.533092292 | 0.000293482 |
| PDHA1 | 0.533070324 | 0.000259934 |
| STAU1 | 0.532882314 | 0.0001995 |
| TENT5C | 0.532878772 | 0.000969916 |
| PDS5A | 0.532876912 | 0.000523843 |
| MMADHC | 0.5327135 | 0.002058798 |
| EIF2AK2 | 0.532666813 | 0.000823593 |
| ZMPSTE24 | 0.532591957 | 0.00178981 |
| AIDA | 0.532509236 | 0.005864427 |
| AMD1 | 0.53218996 | 0.001183498 |
| MORC3 | 0.53206236 | 0.007199068 |
| CD28 | 0.531834812 | 0.045812942 |
| DUSP12 | 0.531659343 | 0.000816212 |
| NPM1 | 0.531496071 | 0.000863769 |
| IFRD2 | 0.531413947 | 0.000199522 |
| PDE4B | 0.531139811 | 0.008889459 |
| CPNE3 | 0.531137987 | 0.003784439 |
| MAP2K1 | 0.530946779 | 0.000750386 |
| USP3 | 0.530863165 | 0.000479528 |
| TTC19 | 0.530775278 | 0.000861399 |
| IFT46 | 0.530744405 | 0.000120468 |
| CERS2 | 0.530391628 | 0.000871948 |
| BZW2 | 0.530193139 | 0.001695382 |
| YWHAQ | 0.530189005 | 0.000861399 |
| MKNK2 | 0.530025296 | 0.000377387 |
| PAM | 0.529720801 | 0.003911465 |
| STARD7 | 0.529459053 | 0.000386474 |
| EIF5B | 0.529458055 | 0.001860446 |
| EIF3H | 0.529409247 | 0.000717024 |
| ST13 | 0.529049665 | 0.001239384 |
| TOR1B | 0.528798426 | 0.000391279 |
| NR1H2 | 0.528784146 | 0.000475017 |
| UNG | 0.528555685 | 0.005395051 |
| CD48 | 0.528366479 | 0.007960203 |
| SREBF2 | 0.528365879 | 0.000167446 |
| BSG | 0.528332205 | 0.002359428 |
| CLN5 | 0.528232564 | 0.004215387 |
| LARP1 | 0.527838754 | 0.00019634 |
| MEF2C | 0.527628229 | 0.001589047 |
| DAP | 0.527498021 | 0.000582634 |
| OSER1 | 0.527494006 | 0.000255199 |
| CRNKL1 | 0.527260153 | 0.013823459 |
| HIGD2A | 0.527143952 | 0.000512149 |
| CALM2 | 0.527105449 | 0.000428518 |
| FAM13B | 0.526864239 | 0.013244503 |
| XPO1 | 0.526804532 | 0.001411637 |
| SPAG1 | 0.526797798 | 0.008735538 |
| BMP8B | 0.526716151 | 0.002032112 |
| ECPAS | 0.526697545 | 0.000179799 |
| EIF3I | 0.526332178 | 0.000370363 |
| POLR2I | 0.526244875 | 0.000473739 |
| UFC1 | 0.526236706 | 0.001443157 |
| GAR1 | 0.525605002 | 0.000360446 |
| IRF1 | 0.525185028 | 0.00608437 |
| SHMT2 | 0.525174054 | 0.007823055 |
| LRFN3 | 0.525148165 | 0.000439824 |
| PBXIP1 | 0.525094314 | 0.002915861 |
| ZNF215 | 0.524822633 | 0.003971899 |
| PRPS1 | 0.524628257 | 0.001629872 |
| PYROXD1 | 0.524217741 | 0.000970542 |
| MFSD6 | 0.524136829 | 0.00732515 |
| ARPP19 | 0.524081816 | 0.002667281 |
| LSM6 | 0.523682721 | 0.002188426 |
| DDX23 | 0.523349062 | 0.000145957 |
| ATP6V1G1 | 0.523282119 | 0.000225866 |
| STAG2 | 0.523263847 | 0.006291088 |
| SMC3 | 0.523242792 | 0.00552566 |
| RNF14 | 0.522177627 | 0.000863769 |
| SRP14 | 0.521670023 | 0.000326718 |
| PARK7 | 0.521641093 | 0.000547486 |
| RSRC2 | 0.521437084 | 0.001014311 |
| IFT20 | 0.521207182 | 0.001629872 |
| PPP3CB | 0.521017513 | 0.000466051 |
| GRHPR | 0.520009771 | 0.000666775 |
| DDX39A | 0.519674307 | 0.003624524 |
| SLK | 0.519589398 | 0.012945112 |
| TSPAN13 | 0.51942799 | 0.027478343 |
| TTC3 | 0.519255868 | 0.00576425 |
| CHMP2A | 0.519098172 | 0.001393438 |
| TICAM1 | 0.518966557 | 0.000108044 |
| MRPL33 | 0.518741039 | 0.000228065 |
| SF3B4 | 0.518636751 | 0.000499224 |
| RPL8 | 0.51858697 | 0.000475527 |
| FCRL2 | 0.518457635 | 0.031471771 |
| UBP1 | 0.517870273 | 0.001450828 |
| DOK3 | 0.517462371 | 0.00291453 |
| BOLA1 | 0.517289297 | 0.001549636 |
| UBE2M | 0.516978133 | 0.002775214 |
| HAT1 | 0.516824182 | 0.008822377 |
| MTO1 | 0.516151258 | 0.001082544 |
| PHB2 | 0.516099621 | 0.001765732 |
| TNRC6B | 0.515435695 | 0.000462077 |
| RNF121 | 0.515048379 | 0.000103705 |
| WBP1L | 0.51497321 | 0.000228065 |
| UQCRQ | 0.514866386 | 0.001589047 |
| SLC25A46 | 0.514749573 | 0.002644331 |
| DHPS | 0.514715313 | 0.001281627 |
| ERH | 0.514409498 | 0.002148467 |
| ROR2 | 0.514260563 | 0.047236169 |
| HYOU1 | 0.513968159 | 0.004621071 |
| STARD5 | 0.513730608 | 0.008496743 |
| PUM1 | 0.513551071 | 0.000238968 |
| TOMM70 | 0.513427377 | 0.001113364 |
| C9orf78 | 0.512849969 | 0.000228065 |
| ALMS1 | 0.512788182 | 0.000871948 |
| PTP4A2 | 0.512579478 | 0.002589134 |
| ECHDC1 | 0.512355964 | 0.00334458 |
| PDIA6 | 0.51217209 | 0.002783551 |
| MRPL3 | 0.512020385 | 0.00284729 |
| CBLL1 | 0.511971785 | 0.005356754 |
| APPL1 | 0.511613636 | 0.003695027 |
| HNRNPA1 | 0.511553935 | 0.000326718 |
| ERGIC2 | 0.511523659 | 0.001948979 |
| RSRP1 | 0.510857381 | 0.010658996 |
| DOLK | 0.510757489 | 0.00026155 |
| ZC3HAV1 | 0.510732738 | 0.000205786 |
| NOP56 | 0.510652396 | 0.0001995 |
| DENND4C | 0.510424963 | 0.005605751 |
| SLC39A7 | 0.510309179 | 0.011365634 |
| TDRD7 | 0.510240851 | 0.003303588 |
| BIK | 0.510067918 | 0.015945373 |
| CMC2 | 0.509861247 | 0.002817624 |
| CTCF | 0.509609219 | 0.000825088 |
| GPD1L | 0.509384939 | 0.004591918 |
| GLO1 | 0.509313277 | 0.005481382 |
| RFC1 | 0.509276067 | 0.002863856 |
| RBM42 | 0.509212549 | 0.000319612 |
| PSMA7 | 0.508949852 | 0.00257795 |
| RSBN1 | 0.508923933 | 0.000411747 |
| FBXW7 | 0.508703287 | 0.005632952 |
| KYAT3 | 0.508701724 | 0.004099724 |
| TMEM109 | 0.508504668 | 0.000899808 |
| SNRPB | 0.508371729 | 0.000590469 |
| UBE2B | 0.508260001 | 0.000103705 |
| MORF4L1 | 0.508115992 | 0.001840013 |
| GALNT12 | 0.50808891 | 0.009972002 |
| CYBC1 | 0.507982411 | 0.001328019 |
| ATP5IF1 | 0.50783599 | 0.000293482 |
| KLF3 | 0.507714235 | 0.003379172 |
| ISCU | 0.507662181 | 0.000973239 |
| DAD1 | 0.507636614 | 0.004264734 |
| GNPAT | 0.507576464 | 0.001410443 |
| EIF1 | 0.507540427 | 0.000103705 |
| DHX15 | 0.50738529 | 0.00180203 |
| PTDSS1 | 0.507281441 | 0.000846305 |
| ORMDL2 | 0.507013344 | 0.002441396 |
| WASHC4 | 0.506941536 | 0.005795222 |
| NCK2 | 0.50689938 | 0.001589537 |
| DNAJC10 | 0.506792021 | 0.005094512 |
| APEX1 | 0.506692191 | 0.002087998 |
| LSM14A | 0.506631481 | 0.00055073 |
| VPS13C | 0.506519586 | 0.000356537 |
| RO60 | 0.506430789 | 0.000527342 |
| TARS1 | 0.50633473 | 0.000127534 |
| HMBS | 0.506259265 | 0.000451972 |
| EAPP | 0.50583444 | 0.007693981 |
| CLCC1 | 0.505464203 | 0.002173735 |
| IQGAP1 | 0.505411003 | 0.004074265 |
| RSF1 | 0.504965142 | 0.002775346 |
| BTN3A2 | 0.504963854 | 0.014665646 |
| FH | 0.504637298 | 0.000927671 |
| AFTPH | 0.504486571 | 0.000613825 |
| SERPINB1 | 0.504430279 | 0.00180203 |
| RWDD1 | 0.503949553 | 0.00047412 |
| KDM3B | 0.503926823 | 0.000750386 |
| ARCN1 | 0.503764902 | 0.001138996 |
| RAPGEF2 | 0.503750873 | 0.009041116 |
| LAX1 | 0.503467744 | 0.030988798 |
| ATP5ME | 0.503406389 | 0.001281627 |
| VEZF1 | 0.502916101 | 0.003565688 |
| TMSB15B | 0.502844293 | 0.00732515 |
| CISD1 | 0.502556761 | 0.006490625 |
| IMMT | 0.50231013 | 0.000477464 |
| PRKD2 | 0.502076218 | 0.00123801 |
| PEA15 | 0.502031866 | 0.007495011 |
| RPL17 | 0.501907516 | 0.000404257 |
| MYG1 | 0.501606591 | 0.002986696 |
| AIP | 0.501578554 | 0.00107219 |
| SLC12A2 | 0.501327258 | 0.00348875 |
| GMPR2 | 0.501259188 | 0.001034647 |
| PSIP1 | 0.501216651 | 0.000514831 |
| RAB11FIP1 | 0.501124514 | 0.015231479 |
| BNIP2 | 0.501050064 | 0.008073229 |
| CDC16 | 0.501031556 | 0.002505702 |
| BMP4 | 0.500715527 | 0.007981553 |
| PTMA | 0.500711417 | 0.000228065 |
| INTS5 | 0.500701619 | 0.000108044 |
| DNAJB9 | 0.5006478 | 0.005813939 |
| TOPBP1 | 0.50059206 | 0.012333723 |
| SCAND1 | 0.500478878 | 0.001068355 |
| MEA1 | 0.500344619 | 0.000364147 |
| ZNHIT3 | 0.500280438 | 0.003379417 |
| H2BC10 | 0.500196973 | 0.003525095 |
| FBXL5 | 0.50010933 | 0.002071386 |
| DCSTAMP | -0.510172926 | 3.21E-05 |
| HOXC8 | -0.512262282 | 0.000145957 |
| C3AR1 | -0.512561537 | 0.007419033 |
| LONRF3 | -0.518223704 | 0.000367157 |
| C1orf105 | -0.522489603 | 0.001532229 |
| IL1R2 | -0.523179749 | 0.000672645 |
| ZBTB16 | -0.523682551 | 0.001969475 |
| CETP | -0.528807821 | 0.0066626 |
| ADGRE1 | -0.532839539 | 0.000543876 |
| CST3 | -0.535629071 | 0.005517165 |
| TGFBR3 | -0.536920003 | 0.00490167 |
| NAP1L3 | -0.551364031 | 0.01387331 |
| CD2 | -0.551436537 | 0.000372161 |
| CCL25 | -0.557003023 | 0.000174775 |
| LPL | -0.558451942 | 0.000860658 |
| HMOX1 | -0.56075996 | 0.047677913 |
| FBXL2 | -0.561660924 | 0.000114282 |
| TEX14 | -0.56614995 | 0.0012074 |
| PAGE1 | -0.568870091 | 0.017993483 |
| CDR1 | -0.573663024 | 0.012496644 |
| TYROBP | -0.581597887 | 0.03655017 |
| IL1B | -0.582177049 | 0.001260284 |
| PCP4 | -0.584275238 | 0.000270763 |
| CD19 | -0.597353254 | 0.035118456 |
| TMEM176A | -0.616470571 | 0.003867318 |
| CFH | -0.626914445 | 0.012070761 |
| AOC1 | -0.628004826 | 0.004329506 |
| IQCG | -0.637485528 | 0.000325891 |
| BNIP3 | -0.700230578 | 0.018676647 |
| CXCL12 | -0.717413873 | 0.03839364 |
| HCAR3 | -0.727860205 | 0.000389376 |
| CXCL8 | -0.733168268 | 0.00157981 |
| PLA2G2D | -0.788979902 | 0.000115666 |
| PRSS21 | -0.808460198 | 0.001434109 |
| CD81 | -0.862277767 | 0.008426899 |
| CTSH | -1.02130591 | 0.006630648 |
| IGLV6-57 | -1.098063669 | 0.000167446 |

| Table S4. Genes selected by the Elastic Net Logistic Regression | | |
| --- | --- | --- |
| 87 genes | | |
| Symbol | Log2FoldChange | adj.P.Val |
| OGT | 1.055208564 | 0.000021 |
| CDC27 | 0.616889073 | 0.0000124 |
| CDKN1A | 0.967495435 | 0.0000216 |
| DCSTAMP | -0.510172926 | 0.0000321 |
| EXOSC5 | 0.695054077 | 0.000103705 |
| BBC3 | 0.598329082 | 0.000103705 |
| PELI1 | 1.130711215 | 0.000103705 |
| RPS21 | 0.614186165 | 0.000103705 |
| RPS27L | 0.793988679 | 0.000108044 |
| FBXL2 | -0.561660924 | 0.000114282 |
| YIPF3 | 0.716978782 | 0.000115344 |
| TARS1 | 0.50633473 | 0.000127534 |
| HOXC8 | -0.512262282 | 0.000145957 |
| IGLV6-57 | -1.098063669 | 0.000167446 |
| GADD45A | 1.186927278 | 0.000167446 |
| CCL25 | -0.557003023 | 0.000174775 |
| H2BC12 | 0.929858892 | 0.000181046 |
| IER2 | 1.107754965 | 0.000216826 |
| ATP6V1G1 | 0.523282119 | 0.000225866 |
| RPL23AP32 | 0.571201677 | 0.000255199 |
| PCP4 | -0.584275238 | 0.000270763 |
| IQCG | -0.637485528 | 0.000325891 |
| HCAR3 | -0.727860205 | 0.000389376 |
| ZFP36 | 0.623590768 | 0.000399565 |
| MTDH | 0.732138292 | 0.000411868 |
| PMEPA1 | 0.782675875 | 0.000428518 |
| KIT | 1.646094978 | 0.000475577 |
| QTRT1 | 0.550728129 | 0.000486884 |
| SF3B4 | 0.518636751 | 0.000499224 |
| ATP1A1 | 0.563207498 | 0.000667389 |
| BTBD3 | 1.630740844 | 0.000690452 |
| PRKCSH | 0.59091127 | 0.000853367 |
| ALMS1 | 0.512788182 | 0.000871948 |
| TMEM268 | 0.57390358 | 0.000990531 |
| MAN2A1 | 0.681426783 | 0.001056662 |
| TOMM70 | 0.513427377 | 0.001113364 |
| ARCN1 | 0.503764902 | 0.001138996 |
| UBE4A | 0.576970532 | 0.001175037 |
| TEX14 | -0.56614995 | 0.0012074 |
| CCDC47 | 0.565485878 | 0.001303001 |
| UFC1 | 0.526236706 | 0.001443157 |
| MEF2C | 0.527628229 | 0.001589047 |
| DHX15 | 0.50738529 | 0.00180203 |
| SERPINB1 | 0.504430279 | 0.00180203 |
| GOLPH3 | 0.543007565 | 0.001809868 |
| UBR7 | 0.536213641 | 0.00187466 |
| ZBTB16 | -0.523682551 | 0.001969475 |
| UGT8 | 0.896164523 | 0.002327467 |
| GALNT3 | 0.572397414 | 0.002394231 |
| SLC35D2 | 0.556855356 | 0.002444393 |
| SLC25A46 | 0.514749573 | 0.002644331 |
| MRPL3 | 0.512020385 | 0.00284729 |
| GAS6 | 0.703830341 | 0.002848164 |
| PRKCB | 0.649550514 | 0.00298176 |
| MYO5C | 0.674516556 | 0.003960657 |
| SLC7A5 | 0.715235694 | 0.004056458 |
| SIDT1 | 0.665682196 | 0.004246344 |
| AOC1 | -0.628004826 | 0.004329506 |
| TGFBR3 | -0.536920003 | 0.00490167 |
| CST3 | -0.535629071 | 0.005517165 |
| GMFG | 0.541418149 | 0.00575474 |
| HSP90AB1 | 0.545122169 | 0.006105415 |
| ALOX5 | 0.556320852 | 0.006234898 |
| C3AR1 | -0.512561537 | 0.007419033 |
| BTAF1 | 0.644417207 | 0.007732604 |
| PTP4A3 | 0.968235359 | 0.007743366 |
| DNAJB14 | 0.557055368 | 0.007838133 |
| CD81 | -0.862277767 | 0.008426899 |
| NECTIN3 | 0.764582875 | 0.008694512 |
| MDK | 0.566915414 | 0.011927657 |
| MFAP3L | 0.56128531 | 0.012077132 |
| CCND1 | 1.237972202 | 0.016174025 |
| TNFSF8 | 0.614846209 | 0.017157579 |
| MYO6 | 0.538694762 | 0.017287792 |
| SERPINI1 | 0.835024687 | 0.017923563 |
| PAGE1 | -0.568870091 | 0.017993483 |
| BNIP3 | -0.700230578 | 0.018676647 |
| SAMSN1 | 0.611660764 | 0.020291196 |
| GGH | 0.672579538 | 0.023482189 |
| HBD | 0.713951069 | 0.033488587 |
| NR4A2 | 0.585120889 | 0.034001616 |
| CD19 | -0.597353254 | 0.035118456 |
| NEB | 0.577615409 | 0.037933227 |
| ERAP2 | 0.6331547 | 0.043014095 |
| CD28 | 0.531834812 | 0.045812942 |
| PTGS2 | 0.716002967 | 0.047519656 |
| CD69 | 0.980806978 | 0.047968706 |

| Supplementary Table S5. Original data for LS-MS/MS analysis | | | |
| --- | --- | --- | --- |
| Fasta header | Charge | Intensity Con | Intensity OSMI-1 |
| HUMAN Cholesterol transporter ABCA5 | 3 | 1330500000 | 0 |
| HUMAN Proliferation-associated protein 2G4 | 2 | 1265600000 | 679750000 |
| HUMAN Tyrosine-protein kinase receptor | 3 | 799440000 | 240470000 |
| HUMAN NBAS subunit of NRZ tethering complex | 3 | 358440000 | 0 |
| HUMAN DOCK4 protein | 4 | 390670000 | 45032000 |
| HUMAN Cytoplasmic FMR1-interacting protein 2 | 3 | 370870000 | 48387000 |
| HUMAN Alpha-fetoprotein | 3 | 255290000 | 0 |
| HUMAN UPF1 | 4 | 374060000 | 160220000 |
| HUMAN Tyrosine--tRNA ligase | 4 | 169620000 | 0 |
| HUMAN Prohibitin | 3 | 501210000 | 361630000 |
| HUMAN Centrosome-associated protein CEP250 | 4 | 138320000 | 0 |
| HUMAN SUMO1/sentrin specific peptidase 5, isoform CRA_a | 2 | 132610000 | 0 |
| HUMAN Histidine utilization repressor | 2 | 265600000 | 142860000 |
| HUMAN Testicular tissue protein Li 111 | 3 | 122280000 | 0 |
| HUMAN E3 ubiquitin-protein ligase TRAF7 | 2 | 160750000 | 40988000 |
| HUMAN Host cell factor 1 | 3 | 118920000 | 0 |
| HUMAN Protein transport protein Sec31B | 3 | 199530000 | 80646000 |
| HUMAN Cullin-9 | 3 | 147300000 | 32810000 |
| HUMAN Nuclear pore complex protein Nup214 | 2 | 153560000 | 41696000 |
| HUMAN Inversin | 3 | 110510000 | 0 |
| HUMAN Ras GTPase-activating protein 3 | 4 | 109570000 | 0 |
| HUMAN Aftiphilin | 3 | 382830000 | 276130000 |
| HUMAN Immunglobulin heavy chain variable region | 3 | 120460000 | 34785000 |
| HUMAN UPF0575 protein C19orf67 | 2 | 123320000 | 37858000 |
| HUMAN Histone-lysine N-methyltransferase 2C | 3 | 190310000 | 109480000 |
| HUMAN Inactive ubiquitin carboxyl-terminal hydrolase 54 | 3 | 79413000 | 0 |
| HUMAN UDP-glucose 6-dehydrogenase | 3 | 114730000 | 37697000 |
| HUMAN HCG2010942 | 3 | 72715000 | 0 |
| HUMAN Break repair meiotic recombinase recruitment factor 1 | 5 | 69216000 | 0 |
| HUMAN Zinc finger protein 726 | 5 | 166860000 | 98000000 |
| HUMAN Rho guanine nucleotide exchange factor 4 | 2 | 66908000 | 0 |
| HUMAN A kinase (PRKA) anchor protein 6, isoform CRA_b | 2 | 81582000 | 14888000 |
| HUMAN UBX domain-containing protein 2B | 4 | 126990000 | 60817000 |
| HUMAN Tektin | 3 | 85674000 | 19788000 |
| HUMAN Glucosidase 2 subunit beta | 2 | 64906000 | 0 |
| HUMAN Immediate early response gene 5-like protein | 3 | 121770000 | 58324000 |
| HUMAN Ryanodine receptor 3 | 3 | 107960000 | 44627000 |
| HUMAN Ryanodine receptor 3 | 3 | 107960000 | 44627000 |
| HUMAN Cysteinyl leukotriene receptor 2 | 3 | 55408000 | 0 |
| HUMAN LEM domain containing 3, isoform CRA_a | 2 | 89441000 | 35080000 |
| HUMAN Zinc finger CCCH domain-containing protein 4 | 3 | 52938000 | 0 |
| HUMAN NEDD4-binding protein 2-like 2 | 3 | 52145000 | 0 |
| HUMAN Ras-responsive element-binding protein 1 | 2 | 107620000 | 56781000 |
| HUMAN Terminal nucleotidyltransferase 5D | 2 | 70732000 | 20043000 |
| HUMAN E3 ubiquitin-protein ligase CBL | 4 | 50382000 | 0 |
| HUMAN Midasin | 3 | 49471000 | 0 |
| _HUMAN IG c1654_heavy_IGHV3-30_IGHD3-10_IGHJ4 | 3 | 118700000 | 72660000 |
| HUMAN IG c1654_heavy_IGHV3-30_IGHD3-10_IGHJ4 | 3 | 118700000 | 72660000 |
| HUMAN Small EDRK-rich factor 1 | 2 | 64675000 | 21184000 |
| HUMAN Protein-associating with the carboxyl-terminal domain of ezrin | 3 | 112590000 | 69623000 |
| HUMAN Mucin 5AC, oligomeric mucus/gel-forming | 2 | 114520000 | 71724000 |
| HUMAN Very large A-kinase anchor protein | 4 | 41041000 | 0 |
| HUMAN Adhesion G-protein-coupled receptor V1 | 4 | 75862000 | 36204000 |
| HUMAN SWI/SNF related, matrix associated, actin dependent regulator of chromatin, subfamily c, member 1 | 3 | 38899000 | 0 |
| HUMAN MHC class II antigen | 3 | 38849000 | 0 |
| HUMAN MHC class I antigen | 4 | 38538000 | 0 |
| HUMAN Nucleolar RNA helicase 2 | 2 | 59621000 | 24103000 |
| HUMAN Cell division cycle protein 27 | 3 | 30166000 | 0 |
| HUMAN RNA helicase | 2 | 50007000 | 20057000 |
| HUMAN Protein mono-ADP-ribosyltransferase PARP12 | 3 | 29189000 | 0 |
| HUMAN Dynein axonemal heavy chain 8 | 2 | 29140000 | 0 |
| HUMAN phosphatidylinositol-3,4-bisphosphate 4-phosphatase | 4 | 28437000 | 0 |
| HUMAN Rho guanine nucleotide exchange factor 2 | 3 | 75441000 | 47064000 |
| HUMAN ST6GALNAC1 protein | 4 | 27568000 | 0 |
| HUMAN RNA-directed DNA polymerase | 3 | 27545000 | 0 |
| HUMAN RNA-directed DNA polymerase | 3 | 27545000 | 0 |
| HUMAN IG c1027_light_IGKV4-1_IGKJ4 | 2 | 27428000 | 0 |
| HUMAN PSMD3 | 2 | 26012000 | 0 |
| HUMAN A-kinase anchor protein 17A | 2 | 25853000 | 0 |
| HUMAN F-box/WD repeat-containing protein 5 | 2 | 104840000 | 80178000 |
| HUMAN Protein phosphatase 1 regulatory subunit | 3 | 24544000 | 0 |
| HUMAN Ectopic P granules protein 5 | 4 | 24489000 | 0 |
| HUMAN Serine/threonine-protein phosphatase with EF-hands 2 | 3 | 45425000 | 21980000 |
| HUMAN Endothelin-converting enzyme 2 | 3 | 22884000 | 0 |
| HUMAN Zinc finger protein 138 | 3 | 35086000 | 12514000 |
| HUMAN Nuclear factor of-activated T-cells, cytoplasmic 1 | 3 | 22316000 | 0 |
| HUMAN Solute carrier family 35 member F5 | 4 | 21571000 | 0 |
| HUMAN Potassium voltage-gated channel subfamily C member 1 | 3 | 21033000 | 0 |
| HUMAN Rap guanine nucleotide exchange factor (GEF) 6 | 2 | 39268000 | 18929000 |
| HUMAN Zinc finger protein 42 (Myeloid-specific retinoic acid-responsive), isoform CRA_a CRA_c OS=Homo sapiens | 3 | 50797000 | 30917000 |
| HUMAN Retinoblastoma-like protein 1 | 2 | 43320000 | 27161000 |
| HUMAN Ribose-phosphate pyrophosphokinase 1 | 3 | 14886000 | 0 |
| HUMAN Oxygen-regulated protein 1 | 3 | 14678000 | 0 |
| HUMAN Kinesin-like protein KIF11 | 2 | 17928000 | 3339100 |
| HUMAN Engulfment and cell motility protein 3 | 3 | 13981000 | 0 |
| HUMAN IGH c1156_heavy_IGHV1-69_IGHD5-12_IGHJ5 | 4 | 36198000 | 23990000 |
| HUMAN LAIR1 | 3 | 11750000 | 0 |
| HUMAN Ras-related protein Rab-3 | 2 | 11386000 | 0 |
